# The RNA binding protein SORBS2 suppresses metastatic colonization of ovarian cancer by stabilizing tumor-suppressive immunomodulatory transcripts

**DOI:** 10.1186/s13059-018-1412-6

**Published:** 2018-03-16

**Authors:** Linjie Zhao, Wei Wang, Shuang Huang, Zhengnan Yang, Lian Xu, Qilian Yang, Xiu Zhou, Jinjin Wang, Qiuhong Shen, Chenlu Wang, Xiaobing Le, Min Feng, Nianxin Zhou, Wayne Bond Lau, Bonnie Lau, Shaohua Yao, Tao Yi, Xin Wang, Xia Zhao, Yuquan Wei, Shengtao Zhou

**Affiliations:** 1 Department of Obstetrics and Gynecology, Key Laboratory of Birth Defects and Related Diseases of Women and Children of MOE and State Key Laboratory of Biotherapy, West China Second University Hospital, Sichuan University and Collaborative Innovation Center, Chengdu, 610041 People’s Republic of China; 2 Department of Biomedical Sciences, City University of Hong Kong, Kowloon Tong, Hong Kong; 3 Department of Pathology, West China Second University Hospital, Sichuan University, Chengdu, People’s Republic of China; 4 College of Life Sciences, Sichuan University, Chengdu, People’s Republic of China; 5 Department of Emergency Medicine, Thomas Jefferson University Hospital, Philadelphia, PA USA; 6 Department of Surgery, Emergency Medicine, Kaiser Santa Clara Medical Center, Affiliate of Stanford University, Santa Clara, CA USA

**Keywords:** RNA binding protein, SORBS2, Ovarian cancer, Metastasis, mRNA stability, Immunomodulation, WFDC1, IL-17D

## Abstract

**Background:**

Ovarian cancer constitutes one of the most lethal gynecologic malignancies for females. Currently, early detection strategies and therapeutic options for ovarian cancer are far from satisfactory, leading to high diagnosis rates at late stages and disease relapses. New avenues of therapy are needed that target key processes in ovarian cancer progression. While a variety of non-coding RNAs have been proven to regulate ovarian cancer metastatic progression, the functional roles of RNA-binding proteins (RBPs) in this process are less well defined.

**Results:**

In this study, we identify that the RBP sorbin and SH3 domain containing 2 (SORBS2) is a potent suppressor of ovarian cancer metastatic colonization. Mechanistic studies show that SORBS2 binds the 3′ untranslated regions (UTRs) of *WFDC1* (WAP four-disulfide core domain 1) and *IL-17D* (Interleukin-17D), two secreted molecules that are shown to act as metastasis suppressors. Enhanced expression of either *WFDC1* or *IL-17D* potently represses SORBS2 depletion-mediated cancer metastasis promotion. By enhancing the stability of these gene transcripts, SORBS2 suppresses ovarian cancer invasiveness and affects monocyte to myeloid-derived suppressor cell and M2-like macrophage polarization, eliciting a tumor-suppressive immune microenvironment.

**Conclusions:**

Our data illustrate a novel post-transcriptional network that links cancer progression and immunomodulation within the tumor microenvironment through SORBS2-mediated transcript stabilization.

**Electronic supplementary material:**

The online version of this article (10.1186/s13059-018-1412-6) contains supplementary material, which is available to authorized users.

## Background

Ovarian cancer has been reported to be the most lethal among gynecologic malignancies, with over 21,000 patients diagnosed and more than 14,000 deaths in the United States in 2014 [1]. The majority of ovarian cancer histological subtypes are high grade serous ovarian carcinoma (HGSOC), with relatively poor prognosis due to the advanced stage of disease at diagnosis, widespread metastasis, and high relapse rate [2]. However, the molecular mechanisms that mediate ovarian cancer progression are far from elucidated, rendering the diagnosis and treatment of ovarian cancer still unsatisfactory.

Ovarian cancer predominantly metastasizes via pelvic dissemination directly to adjacent organs instead of through lymphatic or hematologic channels [3]. Recently, the tumor microenvironment has gradually been recognized to be critical for ovarian cancer intraperitoneal metastasis. Interacting with tumor cells via secretory reciprocal communication, the surrounding microenvironment provides a driving force for cancer cell invasion and metastasis. The stromal cells (including fibroblasts, macrophages, regulatory T cells, myeloid-derived suppressor cells, endothelial cells, pericytes, and platelets), the extracellular matrix (ECM; made of inflammatory cytokines, chemokines, matrix metalloproteinases, integrins, and other secreted molecules), and exosomes (small extracellular vesicles loaded with molecules), which make up the tumor microenvironment, have gained increasing attention due to their vital role in promotion of ovarian cancer progression [4]. Tumor cells educate immune cells in the evolution of different cancer stages [5]. During each step of the metastatic cascade, mutant and thus potentially immunogenic tumor cells are exposed to the immune system, which can recognize them and restrict their growth. However, cancers and their metastatic derivatives could evolve to overcome these immune mechanisms partly through the recruitment of immunosuppressive cells and inactivation of tumor-killing cells [6]. Currently, researchers are focused on how the immune cells are educated, recruited, and/or differentiate to promote cancer metastasis. For instance, Jiménez-Sánchez et al. [7] recently presented an exceptional case of a patient with high-grade serous ovarian cancer, treated with multiple chemotherapy regimens, who exhibited regression of some metastatic lesions with concomitant progression of other lesions during a treatment-free period. Through immunogenomic approaches, they found that progressing metastases were characterized by immune cell exclusion, whereas regressing and stable metastases were infiltrated by CD8+ and CD4+ T cells and exhibited oligoclonal expansion of specific T-cell subsets. They also detected CD8+ T-cell reactivity against predicted neoepitopes after isolation of cells from a blood sample taken almost 3 years after the tumors were resected. These findings suggest that multiple distinct tumor immune microenvironments co-exist within a single individual and may explain in part the heterogeneous fates of metastatic lesions often observed in the clinic post-therapy. Moreover, Montfort et al. [8] recently discovered that B cells mainly infiltrated lymphoid structures in the stroma of HGSOC metastases. There was a strong B-cell memory response directed at a restricted repertoire of antigens and production of tumor-specific IgGs by plasma cells. Chemotherapy could further enhance these responses. These observations highlight the importance of immune cells in intraperitoneal metastasis. However, currently it is still unclear whether RNA binding protein (RBP)-based post-transcriptional regulation of mRNAs could link ovarian cancer metastasis and immune functions.

In this study, we performed an integrated analysis of a HGSOC data set and gene profiles of ovarian cancers to identify key RBPs potentially responsible for ovarian cancer metastatic colonization. We identified the RBP sorbin and SH3 domain containing 2 (SORBS2) as a metastasis suppressor in ovarian cancer. More interestingly, we found that a SORBS2-stablized secretome in ovarian cancer could condition the tumor microenvironment to be favorable for cancer metastasis, via affecting the polarization of monocytes to myeloid-derived suppressor cells (MDSCs) and M2-like macrophages. Therefore, our findings characterize a novel post-transcriptional network that links cancer progression and immunomodulation within the tumor microenvironment through SORBS2-mediated transcript stabilization and could provide a theoretical rational for the potential clinical application value of such therapeutic targets for precision therapy of individuals with ovarian cancer.

## Results

### Integrated analysis identifies SORBS2 as a key RBP that suppresses ovarian cancer metastasis

**Fig. 1:**
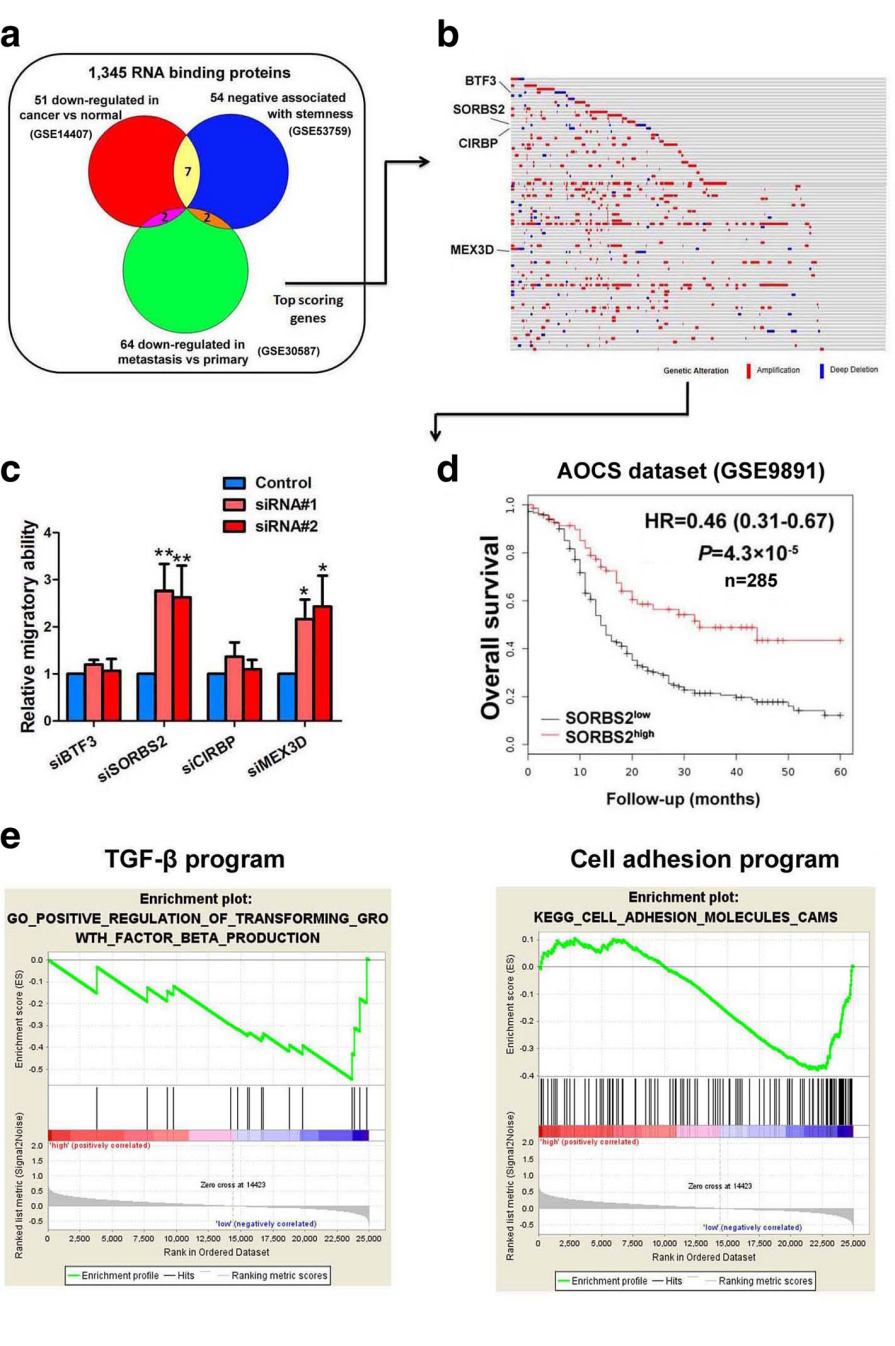
Outline of the screening strategy identifying the RBP SORBS2 as a key suppressor of metastatic colonization of ovarian cancer. **a** Venn diagram outlining the meta-analysis of key RBPs essential for ovarian cancer aggressiveness. **b** Genomic alterations of the top scored RBP genes in TCGA ovarian cancer dataset. **c** Relative migration ability of ovarian cancer cells treated with siRNAs of four significantly downregulated RBPs in TCGA ovarian cancer dataset. **d** Kaplan-Meier analysis of SORBS2 expression and clinical outcome of ovarian cancer in the Australian Ovarian Cancer Study (AOCS) dataset. **e** Gene set enrichment analysis (GSEA) plots showing enrichment of the TGF-β program and the cell adhesion program in different genes between SORBS2 high and SORBS2 low groups in TCGA dataset. Data are shown as mean ± SEM. *P < 0.05, **P < 0.01, ***P < 0.001

To identify key RBPs required for ovarian cancer development and progression, we cross-referenced a list of RBPs in the published literature to compile a comprehensive list of 1345 genes encoding all known human RBPs. Public oncogenomic data were analyzed to score genes based on three properties: (1) lower expression in tumors versus normal tissues (GSE14407) [9]; (2) down-regulated in metastasis sites compared with the primary site of ovarian cancer (GSE30587) [10]; or (3) negative association with the stem-cell state (GSE53759) [11]. Genes scoring in any of these three categories as well as those at the top of each category were selected to define a high-priority set of 145 RBP genes (Fig. 1a and Additional file 1: Table S1). We searched these 145 RBP genes in the cBioPortal TCGA ovarian cancer dataset and examined their amplification and deletion status in these samples. Among all these RBP genes, only four showed deletion in more than 5% of TCGA ovarian cancer samples, including transcription factor BTF3, sorbin and SH3 domain-containing protein 2 (SORBS2), cold-inducible RNA-binding protein (CIRBP), and RNA-binding protein MEX3D (MEX3D) (Fig. 1b). We next screened the functional roles of these four RBPs in ovarian cancer metastasis using a siRNA-mediated RNA silencing strategy in Transwell chamber analysis. We found that only SORBS2 and MEX3D gene knock down significantly increased the metastatic colonization capacity of ovarian cancer (Fig. 1c). Moreover, we searched the prognostic prediction values of these two genes in the Australian Ovarian Cancer Study (AOCS) dataset (GSE9891) and found that only SORBS2 was significantly correlated with overall survival of ovarian cancer patients (Fig. 1d and Additional file 2: Figure S1). In addition, we performed gene set enrichment analysis (GSEA) in TCGA and confirmed that genes comprising the TGF-β program signature and the cell adhesion program signature, two programs widely accepted for their cancer metastasis regulation role, were both highly enriched for ovarian cancer samples with differential SORBS2 expression levels (Fig. 1e), further corroborating the regulatory role of SORBS2 in ovarian cancer metastasis. These data indicate that SORBS2 could be the key RBP that suppresses ovarian cancer metastasis.

### SORBS2 expression is associated with clinical outcome of ovarian cancer patients

We further examined the expression of SORBS2 in different ovarian cancer datasets and found that SORBS2 expression was uniformly down-regulated in ovarian cancer tissues compared with either normal ovary tissues or borderline ovarian tumor tissues in four publicly available datasets (Additional file 2: Figure S2a). Moreover, the expression of SORBS2 in late stage ovarian cancer patients (FIGO stages III and IV) was also significantly reduced compared with early stage ovarian cancer patients (FIGO stages I and II) in Gilks’ dataset and Yoshihara’s dataset (Additional file 2: Figure S2b) while no significant difference was observed in the expression of BTF3, CIRBP, and MEX3D between primary and metastatic ovarian tissues in public datasets (Additional file 2: Figure S3a–c). We next examined the protein expression level of SORBS2 in clinical specimens of ovarian cancer and normal ovary using immunohistochemistry analysis. The results showed that SORBS2 was significantly down-regulated in ovarian cancer compared with normal ovary (Additional file 2: Figure S2c). Moreover, we found that SORBS2 expression was correlated with clinical prognosis in a West China cohort of ovarian cancer (Additional file 2: Figure S2d), consistent with our findings for the AOCS dataset. We further validated our findings in CSIOVDB, a transcriptomic microarray database of 3431 human ovarian cancers that included clinico-pathological parameters and follow-up information of ovarian cancer patients [12]. We observed in the CSIOVDB database that there was significant reduction of SORBS2 expression in ovarian tumors compared with normal ovarian surface epithelium (Additional file 2: Figure S4a). Moreover, CSIOVDB analysis revealed that SORBS2 expression was significantly down-regulated in ovarian cancers with higher differentiation degree (Additional file 2: Figure S4b), more advanced FIGO stage (Additional file 2: Figure S4c), and refractory or resistant disease (Additional file 2: Figure S4d). Consistent with the results from the AOCS and West China cohort, Kaplan-Meier analysis of ovarian cancer patients in CSIOVDB also showed that SORBS2 expression was correlated with overall survival and progression-free survival of ovarian cancer patients (Additional file 2: Figure S4e and Additional file 2: Figure S4f). Moreover, we further analyzed SORBS2 expression with other clinical parameters that might influence the prognosis of ovarian cancer patients in the Tothill dataset (GSE9899) [13], including patient age and disease stage. We found that SORBS2 expression was lower in stage I ovarian cancer patients compared with stage II–IV ovarian cancer patients (Additional file 2: Figure S5a). A negative correlation between age of ovarian cancer patients and SORBS2 was also observed (Additional file 2: Figure S5b).

High grade serous ovarian carcinoma can be classified into four subtypes—immunoreactive subtype, differentiated subgroup, proliferative subgroup, and mesenchymal subgroup [14]—among which the mesenchymal subgroup and the proliferative subtype have poorer prognosis while the immunoreactive subtype and the differentiated subtype have better prognosis [13]. Correspondingly, we have categorized 15 ovarian cancer cell lines into the four subtypes based upon the classifiers used in TCGA ovarian cancer dataset (Additional file 3: Table S2). We performed qRT-PCR to examine the mRNA levels of SORBS2 in these subgrouped ovarian cancer cell lines. Consistently, we found that SORBS2 expression was significantly lower in the group with poorer prognosis (the mesenchymal and proliferative subtypes) compared with the group with better prognosis (the immunoreactive and differentiated subtypes) (Additional file 2: Figure S6a). Moreover, we analyzed the differential expression of SORBS2 in (a) *BRCA1* mutant and *BRCA2* mutant tumor tissues compared with wild-type tumor tissues; (b) CCNE1^high^ and CCNE1^low^ ovarian tumor tissues in TCGA dataset. As shown in Additional file 2: Figure S6b, c, we found that there is a trend that the expression of SORBS2 is lower in *BRCA1* and *BRCA2* mutant (MUT) tissues compared with the *BRCA1* and *BRCA2* wild type (WT) specimens, although no statistical significance was observed (Additional file 2: Figure S6b, c). Moreover, we found a significant negative correlation between the expression of SORBS2 and CCNE1(r = − 0.26, *P* = 8.666e-07; Additional file 2: Figure S6d). The expression of SORBS2 in CCNE1^high^ ovarian cancer specimens is significantly lower compared with that in CCNE1^low^ ovarian cancer specimens (Additional file 2: Figure S6e). These findings indicate that SORBS2 was closely correlated with clinical outcome of ovarian cancer patients.

### SORBS2 suppresses ovarian cancer metastasis in vitro and in vivo

**Fig. 2:**
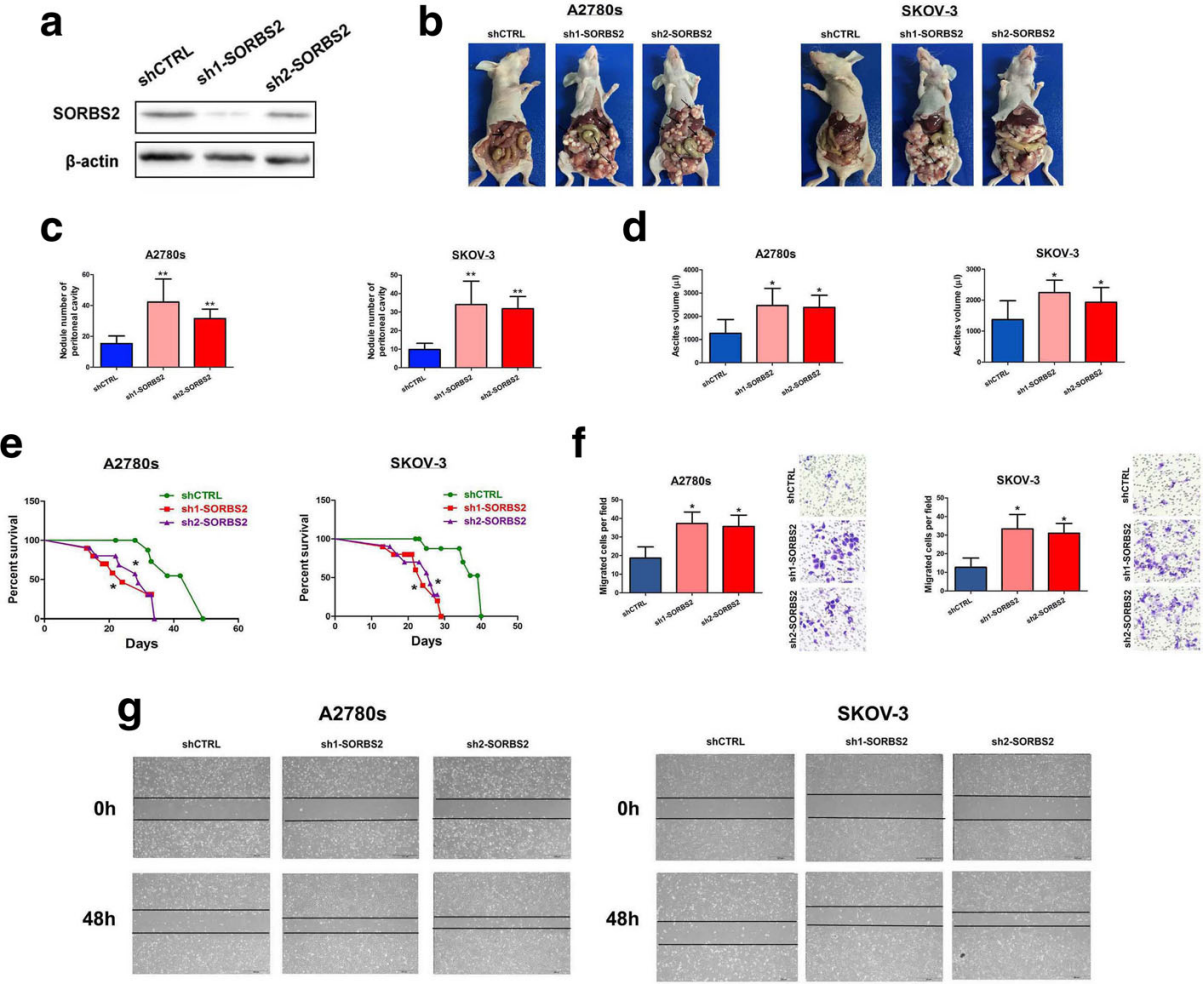
SORBS2 depletion promotes ovarian cancer metastasis. **a** Western blot analysis for SORBS2 in whole-cell lysate of A2780s cells expressing either of two independent shRNAs targeting SORBS2 or a control shRNA. **b** Representative photographs of peritoneal metastasis of A2780s and SKOV-3 ovarian cancer cells 4 weeks post-inoculation. **c** Box plot of number of metastatic nodules of tumors in the abdominal cavities. **d** Box plot of the ascites volumes collected from the abdominal cavities. **e** Kaplan-Meier analysis of mice in the orthotopic model generated by intrabursal injection of either A2780s or SKOV-3 ovarian cancer cells expressing either of two independent shRNAs targeting SORBS2 or a control shRNA. **f** Cell migration capacity of SORBS2-depleted A2780s and SKOV-3 ovarian cancer cells compared with control cells was assessed by transwell analysis. **g** Cell migration capacity of SORBS2-depleted A2780s and SKOV-3 ovarian cancer cells compared with control cells was assessed by wound healing analysis. Data are shown as mean ± SEM. *P < 0.05, **P < 0.01, ***P < 0.001

We next sought to characterize the cellular phenotypes altered in cells depleted of SORBS2. We designed two short-hairpin RNAs (shRNA) to stably silence SORBS2 expression in both SKOV-3 and A2780s ovarian cancer cells (Fig. 2a and Additional file 2: Figure S7a, b). First, we did not observe enhanced primary tumor growth rates in SORBS2 knockdown ovarian cancer cells compared with control cells in vivo (data not shown). Therefore, the enhanced metastatic colonization potential exhibited by SORBS2-knockdown cells is independent of increased proliferation or growth rates. Subsequently, we examined the metastasis-suppressive role of SORBS2 in ovarian cancer in vivo. We used an orthotopic model generated by intrabursal injection in nude mice. The extent of peritoneal metastasis of ovarian cells was examined by sacrifice 4 weeks post-inoculation. We observed that SORBS2 knockdown significantly promoted in vivo metastatic colonization of ovarian cancer cells in mice (Fig. 2b), including increasing the number of metastatic nodules (Fig. 2c) and the ascites volume (Fig. 2d) within the abdominal cavity. Moreover, SORBS2 silencing significantly shortened survival of tumor-bearing mice compared with the control group (Fig. 2e). Immunohistochemical analysis of Ki-67 in tumor tissues in the control group and the SORBS2 knockdown group did not demonstrate significant difference (Additional file 2: Figure S8a), indicating that SORBS2 knockdown primarily hijacked metastatic programs instead of proliferation-related signaling in ovarian cancer. Moreover, we observed a significant decrease in the protein level of cleaved caspase 3 in the lysed tissues of metastasis in the SORBS2 knockdown group compared with that in the control group (Additional file 2: Figure S8b). PI/Annexin V flow cytometry analysis also revealed that the apoptosis rate in the metastasized tissues in the SORBS2-knockdown group was significantly lower than that in the control group (Additional file 2: Figure S8c), revealing an apoptosis resistance-promoting role of SORBS2 knockdown in ovarian cancer.

We further examined the cellular phenotype of SORBS2 depletion in vitro. It was observed that SORBS2 knockdown could induce spindle-like morphology of A2780s cells compared with the original epithelial phenotype (Additional file 2: Figure S8d). Colony formation assay revealed that knockdown of SORBS2 did not affect in vitro cellular proliferation rates (Additional file 2: Figure S8e). Cell cycle analysis by PI staining revealed that while a significant decrease in the percentage of cells in sub G0/G1 phage in the SORBS2 knockdown cell lines compared with control cells, no significant changes were observed in G0/G1, S, and G2/M phase in both SORBS2 knockdown and control cells (Additional file 2: Figure S8f). To define the potential phenotypes displayed by SORBS2-depleted cells that could enhance metastatic activity in vitro, we assessed the ability of SORBS2-knocked down ovarian cancer cells to migrate through Transwell chamber. SORBS2 depletion significantly enhanced migration ability of both A2780s and SKOV-3 ovarian cancer cells through Transwell chamber analysis (Fig. 2f). Consistently, wound healing analysis demonstrated that knockdown of SORBS2 expression in A2780s and SKOV-3 ovarian cancer cells significantly enhanced their metastatic potential (Fig. 2g). These results reveal that SORBS2 impedes the metastasis capacity of ovarian cancer cells in vitro. These findings indicate that reduced expression of SORBS2 is sufficient to promote ovarian cancer metastatic colonization both in vitro and in vivo.

### SORBS2 binds and stabilizes transcripts in ovarian cancer cells

**Fig. 3:**
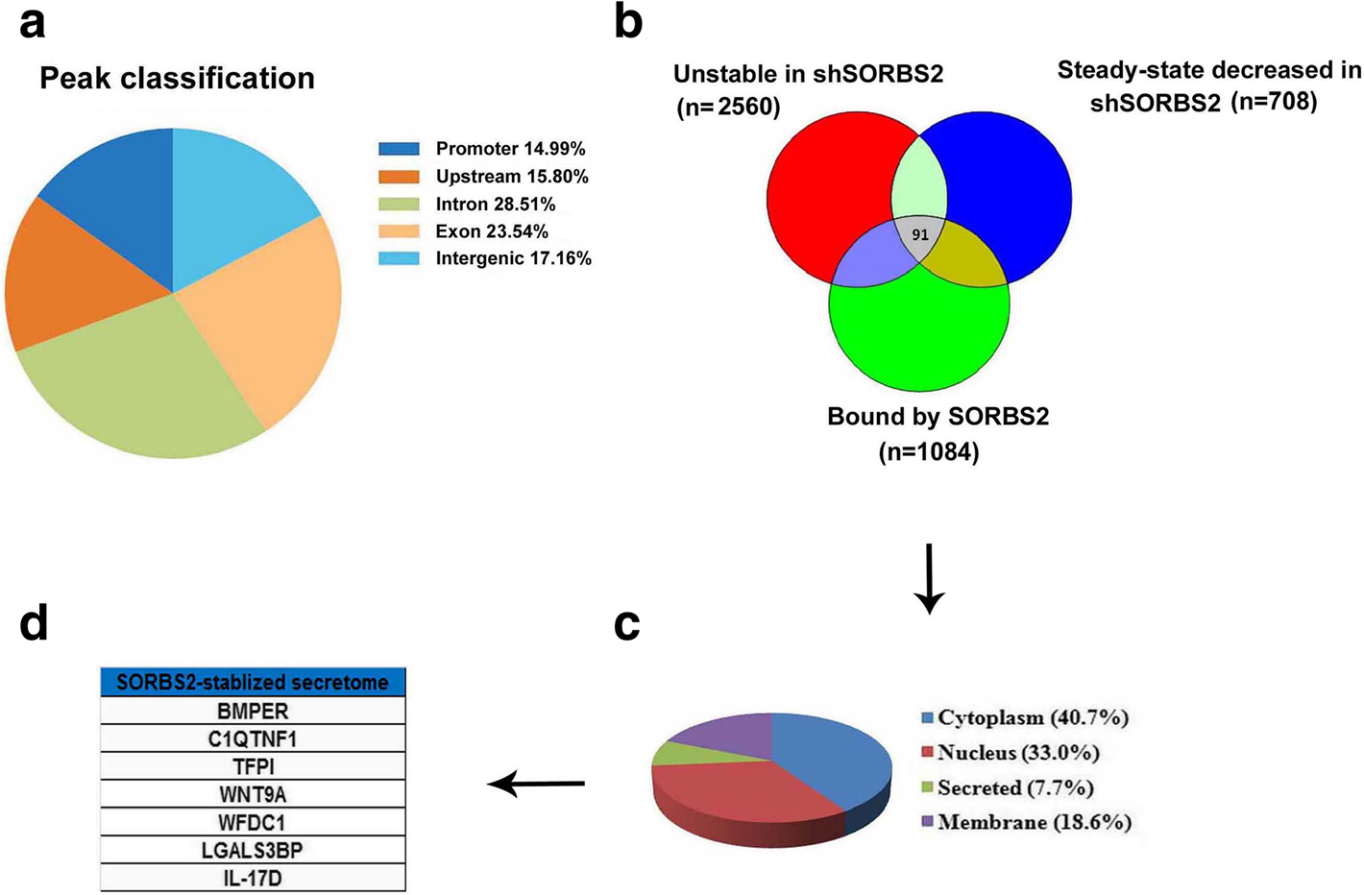
SORBS2 depletion affects the stability of transcripts directly bound by SORBS2. **a** Distribution of SORBS2 RIP sequencing peak annotation for different regions. **b** Venn diagram of transcripts stabilized and bound by SORBS2. Transcripts bound by SORBS2 in the RIP sequencing data (*n* = 1084), transcripts with steady state levels downregulated in shSORBS2 versus control cells (*n* = 708; fold change down > 1.1 in shSORBS2 vs shCTRL, q < 0.05), and transcripts destabilized upon SORBS2 knockdown (*n* = 2560; fold change down > 1.1 after α-amanitin treatment in shSORBS2 vs control cells). **c** Pie chart showing the subcellular locations of proteins translated by SORBS2-stabilized transcripts. **d** Genes encoding secreting proteins translated by SORBS2-stabilized transcripts

We subsequently investigated the molecular mechanisms through which SORBS2 mediates suppression of metastatic colonization of ovarian cancer. Considering that SORBS2 belongs to the RBP family, we attempted to identify its direct targets as potential mediators of its biological effects. First, to identify endogenous RNA targets of SORBS2 in ovarian cancer cells, we performed high-throughput RNA immunoprecipitation sequencing (RIP sequencing) in A2780s ovarian cancer cell lines expressing Flag-tagged SORBS2 and control A2780s ovarian cancer cells (Fig. 3a; Additional file 2: Figure S7c and Additional file 2: Figure S9a–b). Second, considering that one of the most important functions of RBPs is the regulation of RNA stability, we performed transcriptome-wide analysis of RNA stability to identify the RNAs that SORBS2 could stabilize in ovarian cancer metastasis. We treated SORBS2-depleted or control A2780s ovarian cancer cells with α-amanitin and isolated RNA. Relative transcript levels were determined through transcriptomic sequencing, revealing a set of transcripts whose stability was deregulated upon SORBS2 loss. Next, we asked whether transcripts directly bound by SORBS2 were significantly enriched among transcripts with SORBS2-dependent changes in stability. Therefore, we compared the set of SORBS2-bound transcripts (from the RIP-seq data) along with transcript stability measurements obtained from the transcriptomic profiling of SORBS2 depletion and control cells treated with α-amanitin. We found that a proportion of transcripts bound by SORBS2 overlapped with the group of transcripts destabilized by SORBS2 knockdown compared with transcripts with no SORBS2-dependent changes in stability (Fig. 3b). These data demonstrate that SORBS2 could serve as an enhancer of transcript stability through direct binding of transcripts in ovarian cancer. We next attempted to identify SORBS2-bound transcripts that could mediate its effects on ovarian cancer metastasis. As we had observed that SORBS2-bound transcripts were generally stabilized, we sought to identify transcripts bound by SORBS2, destabilized by SORBS2 knockdown, and down-regulated at steady state in SORBS2-depleted cells. Through this analysis, we identified 91 genes (accounting for 8.39% of all the transcripts bound by SORBS2) as potential regulators of ovarian cancer metastasis downstream of SORBS2 (Fig. 3b and Additional file 4: Table S3). Further gene ontology enrichment analysis of biological processes demonstrated that these genes are enriched in biological processes such as regulation of atrial cardiac muscle cell membrane depolarization, kinetochore assembly and organization, metaphase plate congression, establishment of chromosome localization, negative regulation of intrinsic apoptotic signaling pathway in response to DNA damage, and regulation of transcription involved in G1/S transition of mitotic cell cycle (Additional file 5: Table S4). In addition, gene ontology enrichment analysis of KEGG pathways indicated that these genes are enriched in pathways including the FoxO signaling pathway, long-term depression, metabolism of xenobiotics by cytochrome P450, adherens junction, transcriptional misregulation in cancer, proteoglycans in cancer, pathways in cancer, and the cGMP-PKG signaling pathway (Additional file 5: Table S4). These observations indicate that SORBS2 binds to and stabilizes the transcripts in ovarian cancer cells.

### *WFDC1* and *IL-17D* mRNAs are bound by SORBS2 and destabilized by SORBS2 depletion

**Fig. 4:**
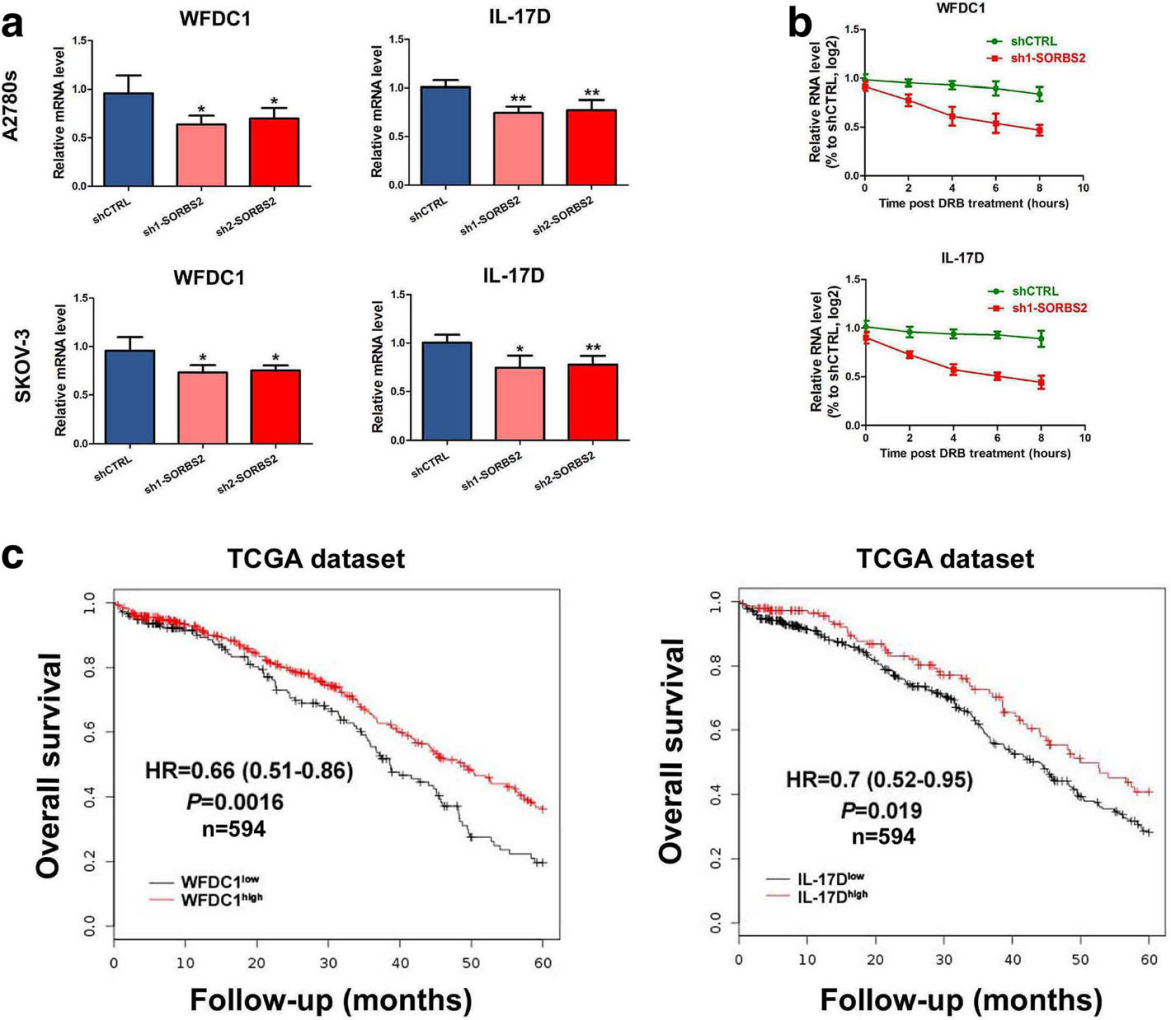
SORBS2 depletion destabilizes transcripts of *WFDC1* and *IL-17D*, which are correlated with clinical outcome in ovarian cancer. **a** qRT-PCR of WFDC1 and IL-17D in control A2780s and control SKOV-3 ovarian cancer cells and SOBRS2-depleted A2780s and SKOV-3 ovarian cancer cells at 0 and 9 h after α-amanitin treatment. 18S was used as an endogenous control. **b** qRT-PCR of WFDC1 and IL-17D in A2780s shSORBS2 and shCTRL cells at the times indicated after treatment of cells with DRB. **c** Kaplan-Meier curve showing overall survival of ovarian cancer patients with tumors expressing high (*red*) or low (*black*) levels of WFDC1 and IL-17D. Data are shown as mean ± SEM. *P < 0.05, **P < 0.01, ***P < 0.001

Among the 91 gene transcripts potentially bound and stabilized by SORBS2, seven transcripts that encode secreted proteins attracted our attention since we focus on potential interactions between ovarian cancer cells and the tumor microenvironment during the metastatic process (Fig. 3c, d). This gene set was comprised of *BMPER*, *C1QTNF1, TFPI, WNT9A, WFDC1, LGALS3BP,* and *IL-17D* (Fig. 3d). The steady-state levels of these seven transcripts were assessed in two SORBS2-knockdown ovarian cancer cell lines and we identified the mRNAs of two genes, *WFDC1* and *IL-17D*, as exhibiting reduced steady-state levels upon SORBS2 knockdown in both cell lines (Additional file 2: Figure S10a, b). The intracellular protein levels of WFDC1 and IL-17D and their relative concentrations in conditioned media were also found to be decreased in SORBS2-depleted cells (Additional file 2: Figure S10e, f). Moreover, we found that SORBS2 binds the 3′ UTRs of *WFDC1* and *IL-17D*, suggesting that SORBS2 may enhance the stability of these transcripts through direct interactions with their 3′ UTRs (Additional file 2: Figure S9c). To further validate this observation, the reduced stability of *WFDC1* and *IL-17D* transcripts upon SORBS2 knockdown was verified by quantitative reverse transcription PCR (qRT-PCR), using α-amanitin to inhibit transcription (Fig. 4a). To validate the stabilization of these two transcripts by SORBS2 using an independent assay of transcription inhibition, we used dichlorobenzimidazole 1-β-D-ribofuranoside (DRB), an inhibitor of CDK9. Following DRB treatment, *WFDC1* and *IL-17D* transcripts exhibited shorter half-lives in SORBS2-depleted cells relative to control cells (Fig. 4b). Moreover, in SORBS2-overexpressing cells, we observed longer half lives of *WFDC1* and *IL-17D* transcripts compared with control cells (Additional file 2: Figure S11a, b). As certain classes of RBPs have a role in modulating poly(A) site selection [15], and considering the location of the SORBS2-binding sites near the 3′ end of the coding sequence stop codons in the *WFDC1* and *IL-17D* transcripts, SORBS2 might regulate the stability of these transcripts through modulation of poly(A) site choice. Therefore, we further examined whether the 3′ UTR lengths of the *WFDC1* and *IL-17D* transcripts were altered in SORBS2-knockdown and in SORBS2-overexpressing cells. We did not find a significant difference in the 3′ UTR lengths of these transcripts upon SORBS2 depletion (Additional file 2: Figure S10c, d). In addition, we did not notice any changes in the 3′ UTR lengths of the *WFDC1* and *IL-17D* transcripts in SORBS2-overexpressing cells compared with control cells (Additional file 2: Figure S11c, d). These results indicates that, in ovarian cancer cells, SORBS2 binds to the 3′ UTRs of *WFDC1* and *IL-17D* transcripts and promotes their stability without affecting the lengths of 3′ UTRs.

### SORBS2-bound targets *WFDC1* and *IL-17D* suppress metastatic colonization in ovarian cancer cells

**Fig. 5:**
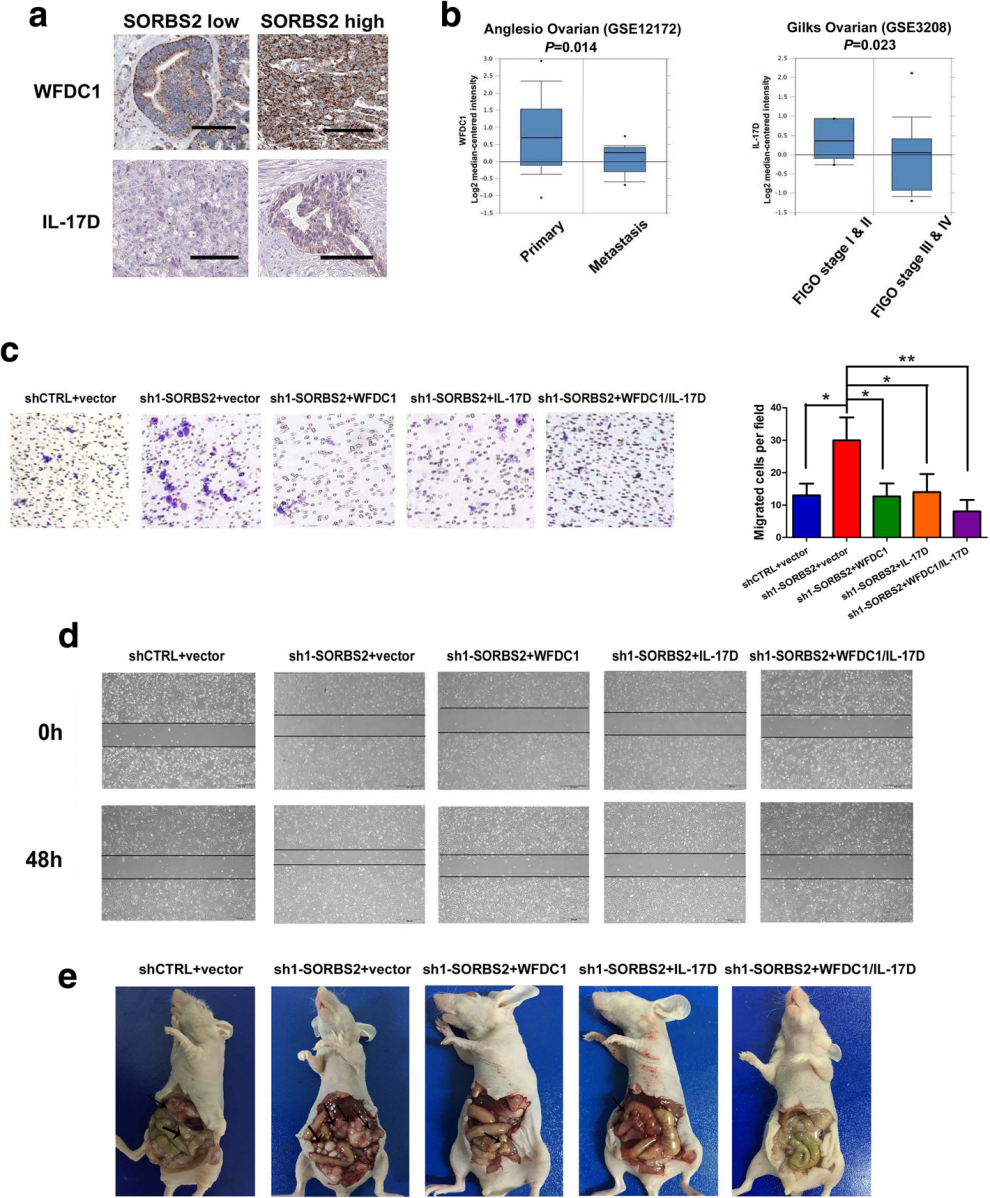
SORBS2 knockdown-induced enhanced ovarian cancer metastasis can be reversed by enforced expression of WFDC1 and IL-17D alone or combined. **a** Immunohistochemistry analysis of WFDC1 and IL-17D expression in SORBS2-low and SORBS2-high metastatic tissues of ovarian cancer. **b** WFDC1 and IL-17D expression at different clinical stages of ovarian cancer in the Oncomine database. **c** WFDC1, IL-17D, and a combination of WFDC1 and IL-17D were stably overexpressed in shSORBS2 A2780s cells. Transwell chamber assays were performed with these cells. **d** WFDC1, IL-17D, and a combination of WFDC1 and IL-17D were stably overexpressed in SORBS2-depleted A2780s cells. Wound healing assays were performed with these cells. **e** Representative photographs of peritoneal metastasis of WFDC1-overexpressed, IL-17D-overexpressed and, WFDC1/IL-17D-overexpressed SORBS2-depleted A2780s ovarian cancer cells compared with control SORBS2-depleted A2780s cells and shCTRL-treated A2780s cells 4 weeks post-inoculation. Data are shown as mean ± SEM. *P < 0.05, **P < 0.01, ***P < 0.001

Given that SORBS2 knockdown reduced the stability and subsequent abundance of *WFDC1* and *IL-17D* transcripts, we attempted to characterize the functional roles of these two genes in metastatic colonization of ovarian cancer cells. We first performed Kaplan-Meier analysis in ovarian cancer patients of TCGA dataset. Results showed that the expression of WFDC1 (*P =* 0.0016, hazard ratio (HR) = 0.66 (0.51–0.86)) and IL-17D (*P =* 0.019, HR = 0.7(0.52–0.95)) was positively correlated with overall survival of ovarian cancer patients in TCGA dataset (Fig. 4c). We next assessed whether the expression of SORBS2 is correlated with the expression of WFDC1 or IL-17D in clinical specimens. Immunohistochemistry analysis revealed that in ovarian cancer specimens with high SORBS2 expression, expression of WFDC1 and IL-17D was also reduced in both primary and metastatic foci of ovarian cancer (Fig. 5a and Additional file 2: Figure S12a). Moreover, we examined the relative concentrations of WFDC1 and IL-17D in the ascites of ovarian cancer patients in low and high SORBS2 tissues. Interestingly, we also observed that the relative concentration of both WFDC1 and IL-17D was significantly lower in SORBS2-low patients compared with that in SORBS2-high patients (Additional file 2: Figure S12b). Oncomine database analysis revealed that in the Anglesio cohort (GSE12172), the expression of WFDC1 was significantly lower in metastatic sites of ovarian cancer compared with primary sites (*P* = 0.014). In addition, in the Gilks cohort (GSE3208), the expression of IL-17D was significantly lower in stage I and II ovarian cancer patients compared with stage III and IV ovarian cancer patients (*P* = 0.023; Fig. 5b).

We next determined whether reconstituting the expression of these genes in cells depleted of SORBS2 could reverse the enhanced metastatic phenotype of ovarian cancer in vitro and in vivo. We observed that stable overexpression of either WFDC1 or IL-17D or a combination of both (Additional file 2: Figure S13a, b) significantly decreased the invasive capacity of SORBS2-depleted ovarian cancer cells compared with control, revealed by Transwell chamber analysis (Fig. 5c) and wound healing analysis (Fig. 5d). Moreover, the in vivo metastatic colonization potential of SORBS2-depleted ovarian cancer cells was also remarkably reduced after overexpression of either WFDC1 or IL-17D or a combination of them (Fig. 5e). In detail, reconstituting the expression of either WFDC1 or IL-17D or a combination of both in SORBS2-knockdown ovarian cancer cells significantly reduced the number of metastatic nodules (Additional file 2: Figure S13c) and ascites volume compared with control SORBS2-knockdown ovarian cancer cells in vivo (Additional file 2: Figure S13d). These findings revealed that overexpression of either WFDC1 or IL-17D or a combination of them is sufficient to repress the metastatic colonization of SORBS2-depleted ovarian cancer cells in vivo as well as suppress migration in vitro.

### SORBS2 recognizes and stabilizes these metastasis-suppressive transcripts via its ZnF_C2H2 domain

**Fig. 6:**
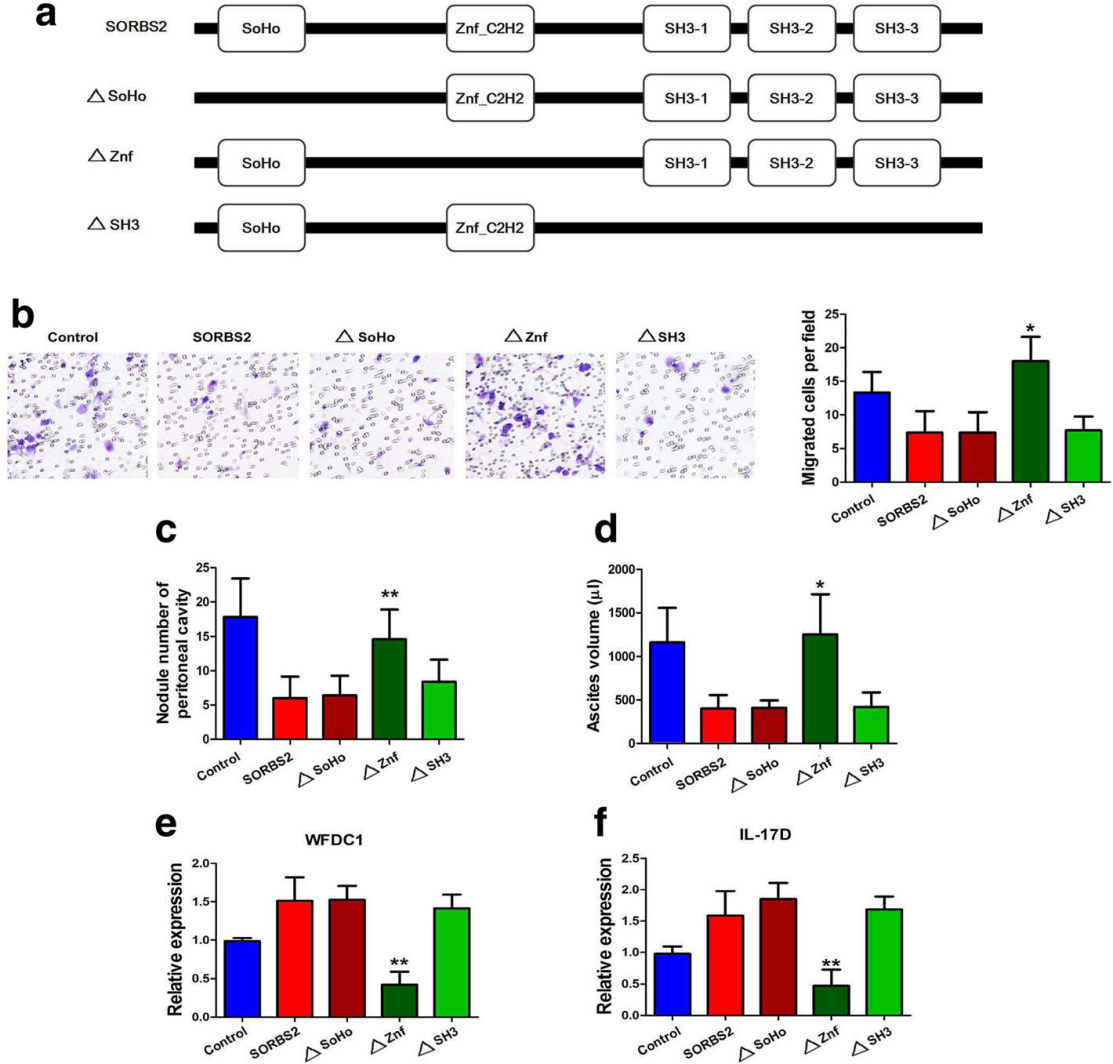
SORBS2 recognizes and stabilizes these metastasis-suppressive transcripts via its ZnF_C2H2 domain. **a** SORBS2 with its binding domains and the respective mutants. **b** Transwell invasion assays were performed in control A2780s cells and A2780s cells with enforced expression of WT SORBS2, ∆SoHo, ∆SH3, and ∆Znf plasmids. **c** Peritoneal metastatic nodule number in mice inoculated with control A2780s cells and A2780s cells with enforced expression of WT SORBS2, ∆SoHo, ∆SH3, and ∆Znf plasmids 4 weeks post-inoculation. **d** Ascites volume in mice inoculated with control A2780s cells and A2780s cells with enforced expression of WT SORBS2, ∆SoHo, ∆SH3, and ∆Znf plasmids 4 weeks post-inoculation. **e** Relative mRNA levels of *WFDC1* in control A2780s cells and A2780s cells with enforced expression of WT SORBS2, ∆SoHo, ∆SH3, and ∆Znf plasmids. **f** Relative mRNA levels of *IL-17D* in control A2780s cells and A2780s cells with enforced expression of WT SORBS2, ∆SoHo, ∆SH3, and ∆Znf plasmids. Data are shown as mean ± SEM. *P < 0.05, **P < 0.01

SORBS2 primarily has three types of domain: one SoHo domain, one ZnF_C2H2 domain, and three SH3 domains. We created three deletion mutants: a SoHo mutant (termed ∆SoHo), containing only the ZnF_C2H2 domain and three SH3 domains and devoid of the SoHo domain; a Znf mutant (termed ∆Znf), containing only the SoHo domain and three SH3 domains and lacking the ZnF_C2H2 domain; a SH3 mutant (termed ∆SH3), containing only the SoHo domain and one ZnF_C2H2 domain and devoid of the three SH3 domains (Fig. 6a). Human ovarian cancer cell line A2780s was transfected with wild-type (WT) SORBS2, ∆SoHo, ∆Znf, and ∆SH3 plasmids to determine which domain is key for SORBS2 to recognize and stabilize the abovementioned transcripts. Interestingly, we found that enforced expression of ∆SoHo and ∆SH3 plasmids significantly suppressed ovarian cancer migration in vitro compared to WT SORBS2 protein while enforced expression of ∆Znf plasmids did not inhibit ovarian cancer cell migration (Fig. 6b). Moreover, we observed significantly decreased numbers of metastatic nodules and reduced volume of ascites of mice inoculated with A2780s cells with enforced expression of ∆SoHo and ∆SH3 plasmids, similar to A2780s cells with enforced expression of WT SORBS2. By contrast, no significant reduction in the number of metastatic nodules and ascites volume in mice inoculated with A2780s cells with enforced expression of the ∆Znf plasmid was observed compared with control A2780s cells (Fig. 6c, d). In addition, we determined whether the ZnF_C2H2 domain was indispensable for SORBS2-mediated stabilization of *WFDC1* and *IL-17D*. We found that the expression level of both WFDC1 and IL-17D in A2780s cells with enforced expression of ∆SoHo and ∆SH3 plasmids was comparable to that in A2780s cells with enforced expression of WT SORBS2. However, both WFDC1 and IL-17D expression levels were remarkably decreased in A2780s cells with enforced expression of the ∆Znf plasmid (Fig. 6e, f). These data indicate that, among the three domains, only the ZnF_C2H2 domain is functional in binding to and stabilizing target mRNAs by SORBS2.

### SORBS2 depletion-induced secretome alterations are associated with monocyte to MDSC and M2-like macrophage polarization

**Fig. 7:**
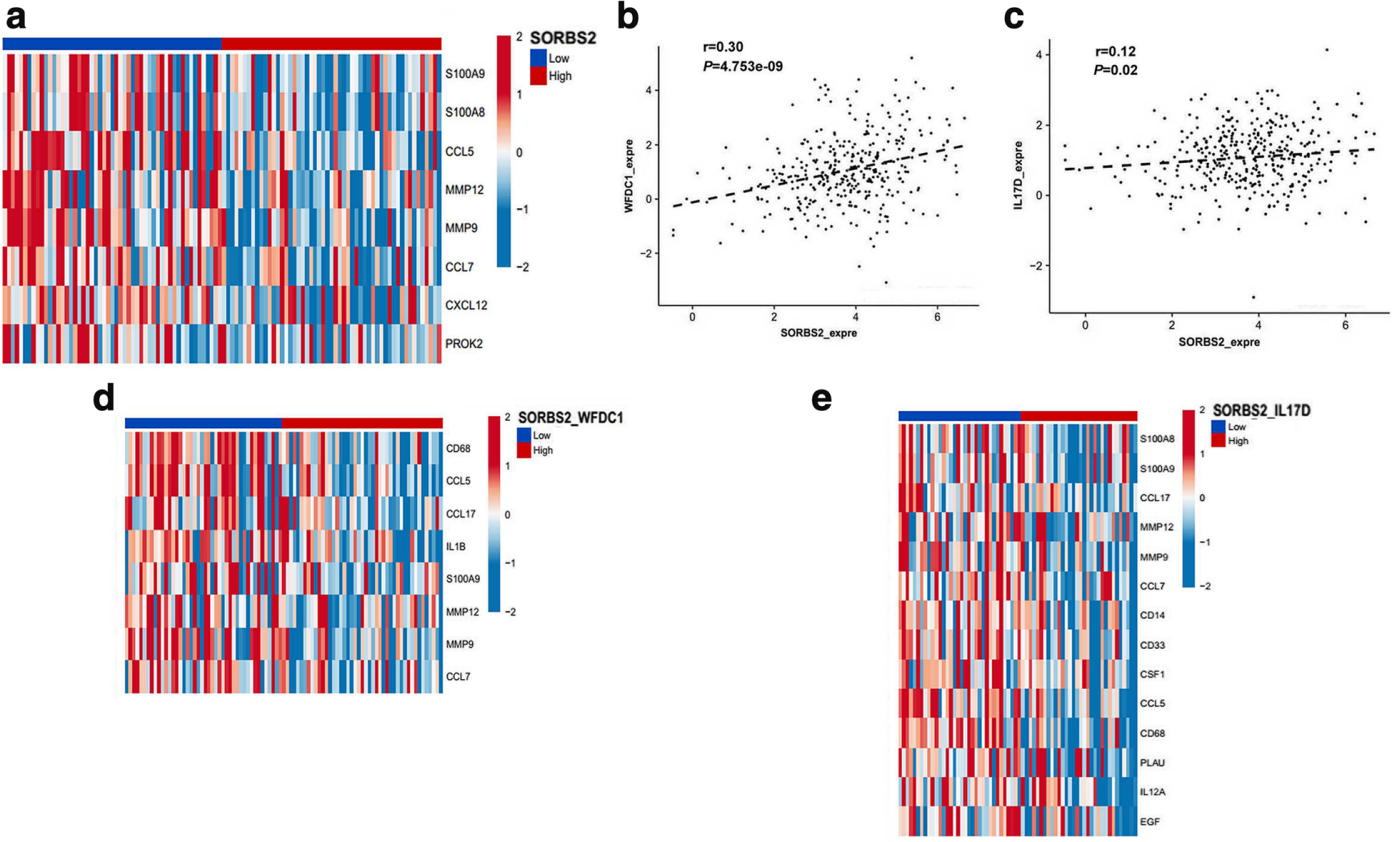
WFDC1 and IL-17D correlate with a tumor-suppressive immune profile in ovarian cancer patients. **a** Heatmap of immune-related genes significantly co-expressed (*P* = 0.01) with SORBS2, showing log2 expression Z scores for 20% of samples with highest or lowest SORBS2 expression. **b** Correlation data for SORBS2 versus WFDC1 expression in TCGA dataset. The statistical significance of correlations was determined using Pearson’s correlation coefficient. **c** Correlation data for SORBS2 versus IL-17D expression in TCGA dataset. The linear regression curve is shown as a black line for significant correlations. **d** As in **a** for 50% of samples with the highest or lowest combined SORBS2 and WFDC1 expression. **e** As in **b** for samples with highest or lowest combined SORBS2 and IL-17D expression

As WFDC1 and IL-17D are secreted factors released by SORBS2-low ovarian cancer cells, we wondered whether they have any impact on the tumor microenvironment upon ovarian cancer metastasis. Specifically, we focused on the pro-cancer immunomodulatory role of SORBS2 and its bound targets *WFDC1* and *IL-17D*. RNA sequencing (RNA-seq) data from a cohort of 354 high grade serous ovarian carcinoma (HGS-OvCa) patients (Cancer Genome Atlas Research Network, 2014), obtained from TCGA (https://cancergenome.nih.gov/), to bioinformatic co-expression analysis focusing on immune cell markers and cytokines. Interestingly, SORBS2 expression showed a significant negative correlation with the expression of 11 M2 myeloid cell markers and cytokines associated with their expansion (Fig. 7a). Next, we determined whether an association exists between the expression of SORBS2 and the expression of WFDC1 or IL-17D in TCGA dataset. We found a positive correlation between SORBS2 expression and WFDC1 expression (Fig. 7b) as well as between SORBS2 expression and IL-17D expression (Fig. 7c). We further analyzed combined SORBS2 and WFDC1 high versus low expression levels as well as SORBS2 and IL-17D high versus low expression levels with respect to co-expression with factors involved in M2-like cell signaling. Corresponding with a decisive role for SORBS2-stablized *WFDC1* and *IL-17D* in generating a metastasis-suppressing phenotype of ovarian cancer, the expression levels of 8 and 14 M2 markers and cytokines also showed significant negative correlation with combined SORBS2/WFDC1 (Fig. 7d) and SORBS2/IL-17D levels (Fig. 7e), respectively, constituting a tumor-suppressive immune microenvironment.

**Fig. 8:**
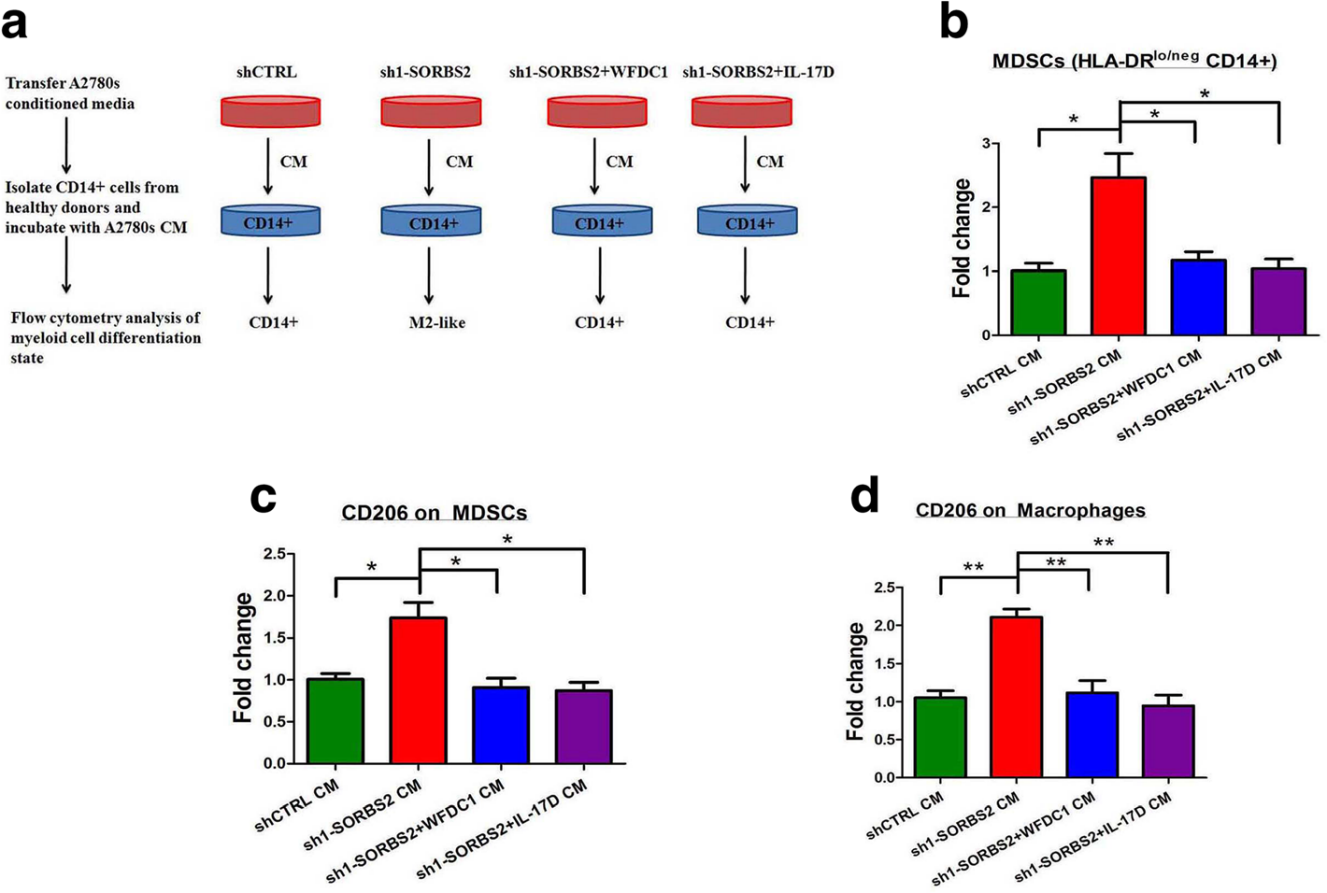
SORBS2 depletion-induced secretome alterations are associated with monocyte to MDSC and M2-like macrophage polarization in human. **a** A schematic model of the monocyte polarization protocol. CD14+ cells were isolated from healthy donor peripheral blood mononuclear cells (PBMCs) via magnetic CD14+ microbeads and incubated with conditioned medium (*CM*) from control A2780s ovarian cancer cells, SORBS2-depleted A2780s ovarian cancer cells, WFDC1-overexpressing SORBS2-depleted A2780s ovarian cancer cells, and IL-17D-overexpressing SORBS2-depleted A2780s ovarian cancer cells. After 48 h, treated myeloid cells were stained with fluorochrome-labeled antibodies against HLA-DR, CD14, and CD206 and analyzed by flow cytometry. Data are presented as fold change in HLA-DRlo/neg CD14+ cells (**b**), HLA-DRlo/neg, CD14+, CD206+ cells (**c**), or HLA-DR+, CD14+ CD206+ macrophages (**d**) upon conditioned media incubation. Data are shown as mean ± SEM. *P < 0.05, **P < 0.01, ***P < 0.001

In this context, we further investigated whether the SORBS2 depletion-induced secretome might functionally affect immune cell polarization. To this end, human healthy donor CD14+ cells were cultured with conditioned media of control or SORBS2-knockdown A2780s ovarian cancer cells (Fig. 8a). Interestingly, conditioned media of SORBS2-depleted A2780s cells polarized healthy donor CD14+ cells toward an HLA-DRlo/neg phenotype, an immune cell population equivalent to murine CD11b + GR1+ MDSCs, compared with conditioned media of control A2780s cells. However, conditioned media from either WFDC1 or IL-17D overexpressed, SORBS2-depleted ovarian cancer cells significantly reduced the amount of HLA-DRlo/neg CD14+ cells (Fig. 8b). Furthermore, HLA-DRlo/neg as well as HLA-DR+ cells displayed increased levels of CD206 expression after incubation of conditioned media of SORBS2-depleted A2780s cells compared with conditioned media of control A2780s cells, indicating polarization toward MDSCs and fully differentiated M2 macrophage phenotypes, respectively (Fig. 8c, d). By contrast, conditioned media from either WFDC1 or IL-17D overexpressed SORBS2-depleted ovarian cancer cells could obviously reverse this process (Fig. 8c, d). Therefore, SORBS2 depletion-induced secretome alterations are associated with monocyte to MDSC and M2-like macrophage polarization, which indicates an immunomodulatory role of SORBS2 and its stabilized transcripts in the tumor microenvironment.

### Cancer-derived SORBS2-stabilized secretome suppresses tumor metastasis and recruitment of tumor-supportive infiltrates in vivo

As the full extent of the effect of the cancer-derived SORBS2-stabilized secretome on immune cell infiltration can most suitably be assessed in immune-proficient mice, we next made use of the established ID-8 ovarian cancer peritoneal metastasis mouse model. We first established a SORBS2-knockdown ID-8 cell line, WFDC1-overexpressing SORBS2-knockdown ID-8 cell line, and IL-17D-overexpressing SORBS2-knockdown ID-8 cell line. The transfection efficacy was evaluated by measuring the mRNA levels of each gene (Additional file 2: Figure S14a). We next performed intrabursal injection of these cell lines in C57BL/6 immune-competent mice. At sacrifice, we found that the SORBS2-knockdown ID-8 cell group demonstrated significantly more metastatic nodules (Additional file 2: Figure S14b, c) and ascites volume (Additional file 2: Figure S14d, e) compared with the control group. Moreover, reconstituting the expression of either WFDC1 or IL-17D in the SORBS2-knockdown ID-8 cell group significantly reduced the number of metastatic nodules (Additional file 2: Figure S14b, c) and ascites volume (Additional file 2: Figure S14d, e) compared with the SORBS2-knockdown ID-8 cell group. These findings are consistent with our observations in immune-deficient mouse models.

**Fig. 9:**
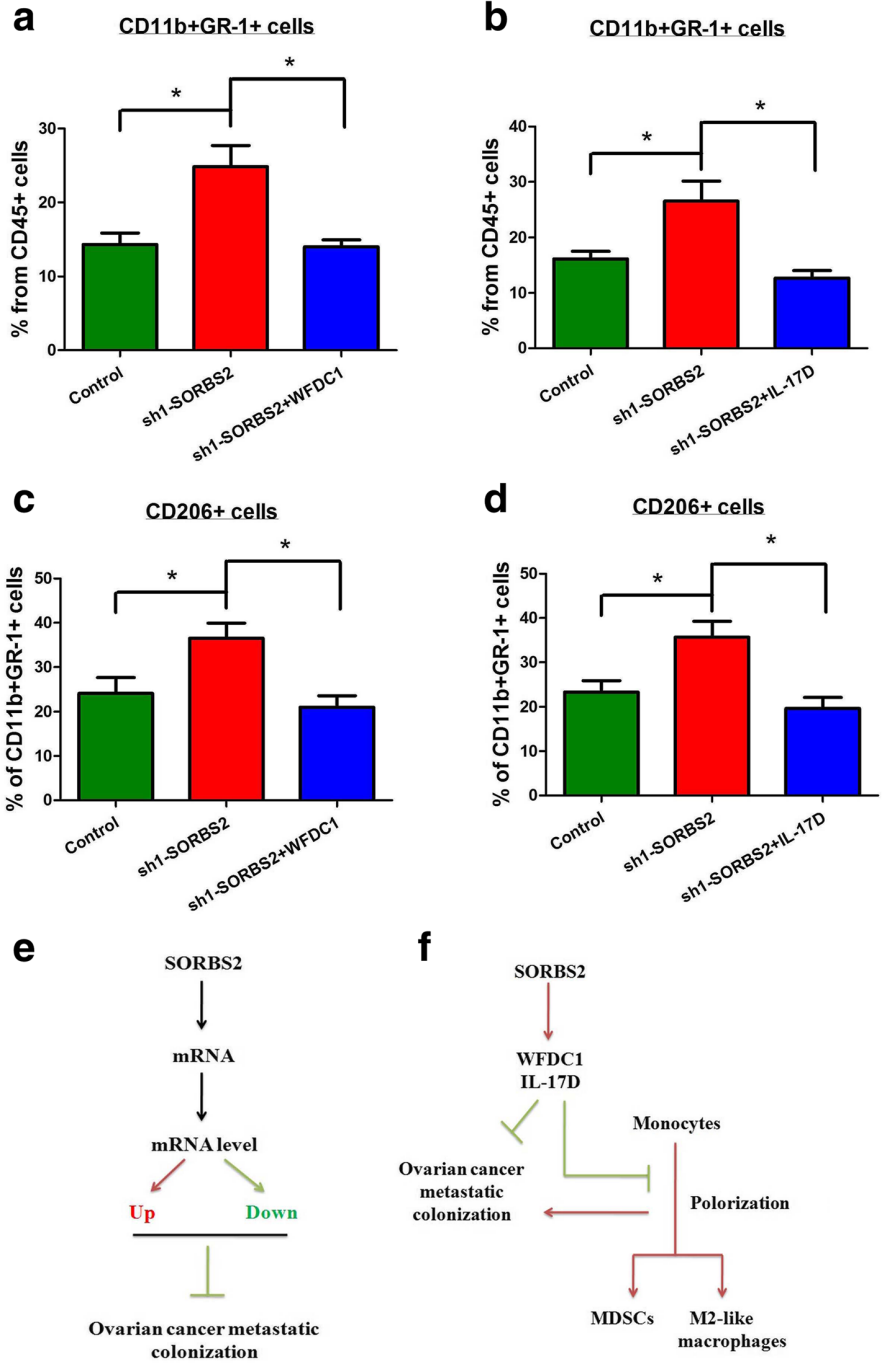
Cancer-derived SORBS2-stabilized secretome suppresses tumor metastasis and recruitment of tumor-supportive infiltrates in vivo. **a** The percentage of CD11b + GR-1+ cells in the CD45+ cells of the metastatic nodules of C57BL/6 mice intrabursally inoculated with control ID-8 cells, SORBS2-knockdown ID-8 cells, and WFDC1 overexpressing SORBS2-knockdown ID-8 cells. *n* = 6 in each group. **b** The percentage of CD11b + GR-1+ cells in the CD45+ cells of the metastatic nodules of C57BL/6 mice intrabursally inoculated with control ID-8 cells, SORBS2-knockdown ID-8 cells, and IL-17D-overexpressing SORBS2-knockdown ID-8 cells. *n* = 6 in each group. **c** The percentage of CD206+ cells in the CD11b + GR-1+ cells of the metastatic nodules of C57BL/6 mice intrabursally inoculated with control ID-8 cells, SORBS2-knockdown ID-8 cells, and WFDC1-overexpressing SORBS2-knockdown ID-8 cells. n = 6 in each group. **d** The percentage of CD206+ cells in the CD11b + GR-1+ cells of the metastatic nodules of C57BL/6 mice intrabursally inoculated with control ID-8 cells, SORBS2-knockdown ID-8 cells, and IL-17D-overexpressing SORBS2-knockdown ID-8 cells. n = 6 in each group. **e** At the global level, SORBS2 could bind different kinds of mRNAs and stabilize a proportion of these mRNAs. The net effect of progression-promoting and -inhibiting alterations determine whether SORBS2 loss is beneficial for ovarian cancer metastatic colonization. **f** At the target mRNA level, SORBS2 could stabilize the transcripts of WFDC1 and IL-17D, which leads to overexpression of these secreting factors. On one hand, they could partly suppress ovarian cancer metastasis; on the other hand, they could inhibit the polarization of monocytes towards MDSCs and M2-like macrophages, which is important for a immune suppressive tumor microenvironment favorable for ovarian cancer metastatic colonization. Data are shown as mean ± SEM. *P < 0.05, **P < 0.01, ***P < 0.001

In line with a functional involvement of alternatively activated myeloid cells in influencing tumor burden, the number of CD11b + GR1+ myeloid infiltrates within the tumor tissue of the SORBS2-knockdown group were significantly increased compared with those in the control ID-8 group (Fig. 9a, b). Moreover, the expression of CD206 on these myeloid infiltrates also significantly increased in the SORBS2-knockdown group compared with the control ID-8 group (Fig. 9c, d). However, reconstituting the expression of either WFDC1 or IL-17D in the SORBS2-knockdown ID-8 cell group significantly reduced the content of CD11b + GR1+ myeloid infiltrates as well as the expression of CD206 in these myeloid infiltrates compared with those in the SORBS2-knockdown ID-8 cell group (Fig. 9a–d). Thus, we conclude that the cancer-derived SORBS2-stabilized secretome could potently suppress ovarian cancer metastasis and modulate the accumulation of tumor-promoting myeloid cells in vivo (Fig. 9e, f).

## Discussion

Surgical cytoreduction of advanced-stage ovarian cancer has long been considered an important tenet of effective management of this disease. Although the sequence of chemotherapy and surgical intervention is debated, there is broad consensus that integration of the two modalities represents the best initial strategy for women with metastatic ovarian cancer [1, 16]. So far, the molecular mechanisms underlying the metastatic process of ovarian cancer remain largely unknown. In this study, we used a combination of computational, biochemical, and functional approaches to identify key molecular events that underly the metastatic process of ovarian cancer and characterize a novel post-transcriptional network that controls ovarian cancer metastatic colonization through SORBS2-mediated transcript stabilization.

Precise regulation of RNA metabolism is instrumental to the generation of biological complexity in both normal and pathological circumstances. The concerted action of RBPs regulate the spatial, temporal, and functional dynamics of the transcriptome via alternative splicing, alternative polyadenylation, and transcript stability [17]. While dysregulation of RNA metabolism via abnormal microRNA expression is relatively well established, accumulating evidence indicates a vital role also for RBPs in the development and progression of cancer. However, the functional role of this group of proteins in ovarian cancer has not been reported. In this study, we elucidate a mechanism through which the RBP SORBS2 mediates its suppressive effect in ovarian cancer metastatic colonization. SORBS2 enhances the stability of the transcripts by binding to the 3′ UTRs of target mRNAs, without influencing the lengths of those 3′ UTRs. SORBS2, also known as Arg/c-Abl kinase binding protein 2 (ArgBP2), is an adapter protein that could interact with multiple actin regulatory proteins, including Arg, c-Abl, Vinculin, Paxillin, WAVE, c-Cbl, Pyk2, PKB, PAK1, α-actinin, 14–3-3, and SAPAP, which can be generally grouped into cell adhesion molecules and regulators and effectors of small GTPases [18]. Previous research has highlighted its functional role in disease states, such as intellectual disability, gastric cancer, sepsis-associated cardiac dysfunction, and facioscapulohumeral dystrophy [19–22]. However, its role as an RBP has rarely been reported. Like other RBPs which either directly modulate transcript stability or regulate alternative splicing patterns, SORBS2 could impact gene expression and phenotypic output of ovarian cancer cells in part through enhancing the stability of a subset of gene transcripts, especially the metastasis suppressor *WFDC1* and *IL-17D* transcripts, and interacting with their 3′ UTRs.

*WFDC1* and *IL-17D* are examples of SORBS2-bound transcripts that are directly regulated by SORBS2 and act as metastasis suppressors. WAP four-disulfide core domain 1 (WFDC1) is a 24 kDa secreted protein encoded by the WFDC1 gene [23]. It is part of the WAP domain-containing family of proteins consisting of small secreted immunomodulatory factors that are being increasingly recognized as important regulators of cell and tumor growth. Previous reports have demonstrated that WFDC1 expression was dramatically downregulated in highly prolific mesenchymal cells and in a number of cancers, including fibrosarcomas, and in tumors of the lung, bladder, prostate, and brain [24]. Moreover, overexpression of WFDC1 inhibited the growth rate of the fibrosarcoma HT1080 cell line. In line with these findings, our study also proved that overexpression of WFDC1 in SORBS2-depleted ovarian cancer cells significantly impaired their metastatic potential. IL-17D, which belongs to the IL-17 family of cytokines, is a cytokine whose function is not well annotated. Although similar to IL-17C, it is known to be expressed outside the immune system and could stimulate human umbilical vein endothelial cells to produce IL-6, IL-8, and GM-CS [25]. Recently, its role in tumor development and progression has been investigated. O’Sullivan et al. [26] reported that IL-17D was highly expressed in certain unedited tumors but not in edited mouse tumor cell lines. Moreover, forced expression of IL-17D in edited tumor cells induced rejection by stimulating MCP-1 production from tumor endothelial cells, resulting in the recruitment of natural killer (NK) cells. NK cells promoted M1 macrophage development and adaptive immune responses. IL-17D expression was also decreased in certain high-grade and metastatic human tumors, suggesting that it can be targeted for tumor immune therapy. Our study demonstrated that forced expression of IL-17D in SORBS2-depleted ovarian cancer cells could also significantly reduce ovarian cancer metastatic colonization. Thus, SORBS2-bound transcripts of *WFDC1* and *IL-17D* served as potent metastatic suppressors in ovarian cancer.

As WFDC1 and IL-17D are secreted factors and are reported to be immunomodulatory, we further focused on the immunomodulatory role of these two genes in ovarian cancer metastasis. Ressler et al. [27] showed that Wfdc1-null mice infected with influenza A exhibited 2.75-log-fold lower viral titer relative to control mice. Wfdc1-null infected lungs exhibited elevated macrophage levels and deposition of osteopontin, a potent macrophage chemokine. In wounding studies, Wfdc1-null mice exhibited an elevated rate of skin closure, and this too was correlated with elevated deposition of osteopontin and macrophage recruitment. Wfdc1-null fibroblasts exhibited impaired spheroid formation, elevated adhesion to fibronectin, and an increased rate of wound closure in vitro. This was reversed by neutralizing antibody to osteopontin. These data suggest that WFDC1 is a core component of the immunomodulatory network. Moreover, recent research has also recognized the key position of IL-17D in modulating the immune system. Apart from O'Sullivan et al.’s observation that IL-117D mediates tumor rejection through recruitment of natural killer cells, Saddawi-Konefka et al. [28] also demonstrated that the transcription factor nuclear factor erythroid-derived 2-like 2 (Nrf2) could induce the expression of IL-17D in cancer cell lines. Expression of IL-17D in tumors and virally infected cells is essential for optimal protection of the host as il17d(−/−) mice experienced a higher incidence of tumors and exacerbated viral infections compared to WT animals. Moreover, activating Nrf2 to induce IL-17D in established tumors led to NK cell-dependent tumor regression. These data demonstrate that IL-17D effects a form of extrinsic stress surveillance by inducing antitumor immunity. Our study shows that supernatants of both WFDC1- and IL-17D-overexpressed, SORBS2-depleted ovarian cancer cells significantly reduced the amount of HLA-DRlo/neg CD14+ cells in vitro. Moreover, in an immuno-proficient model, we also found that WFDC1 and IL-17D could potently suppress ovarian cancer metastasis and are able to inhibit the accumulation of tumor-promoting myeloid cells. Therefore, it is conceivable that *WFDC1* and *IL-17D*, as part of a SORBS2-stablized secretome, could play a crucial role in modulating the polarization of myeloid cells within the tumor microenvironment, which remodel it towards a tumor-suppressive immune milieu.

## Conclusions

Our study illustrates a novel RBP-based post-transcriptional network that links cancer progression and immunomodulation within the tumor microenvironment. The transcripts, in particular *WFDC1* and *IL-17D*, encode secreting factors that not only limit the metastasis of ovarian cancer, but also remodel the tumor immune microenvironment towards tumor suppression. Our findings have identified a functional role of SORBS2 and its stabilized transcripts in ovarian cancer progression, deepening our understanding of the molecular complexities of ovarian cancer metastasis.

## Methods

### Bioinformatics analysis

As a starting point for identifying RBPs required for ovarian cancer aggressiveness, we first collected a list of 1345 genes encoding RBPs from high-throughput screens by Castello et al. [29], Baltz et al. [30], and Ray et al. [31], human orthologs of RBPs identified in mouse embryonic stem cells by Kwon et al. [32], and RBPs reported in RBPDB [33]. Proteins annotated in ENSEMBL’s human genome build which were not identified as RBPs were considered as non-RBPs. To generate a cancer-relevant “high priority” subset of RBP genes (out of the 1345 genes we classified as RBPs), we first identified those genes whose expression are significantly associated with the cancerous state, stemness, or metastasis in ovarian cancer. Genes associated with the cancerous state were obtained by analyzing the differential gene expression that profiled normal ovary tissues and primary ovarian tumors in GSE14407 [9]. Genes associated with stemness were obtained by analyzing the different gene profiles between ovarian cancer spheroids and ovarian cancer monolayers in GSE53759 [11]. To identify the genes associated with metastasis, we analyzed the differential gene expression profiles between nine matched pairs of primary ovarian tumors and metastases from the omentum in GSE30587 [10]. In each dataset, the gene expression profiles were classified between the two groups and for each group the log2 median centered intensity for each gene was determined. A *P* value associated with the significance of the difference between the two groups was calculated with the Student’s *t*-test. From all these genes with a *P* value< 0.05, we identified those that are in the top 10% of the most down-regulated RBP genes in each dataset. To generate the final high priority set of 145 genes that was screened, three categories of genes were selected: (1) genes scoring in all three analyses; (2) the most significantly scoring 5% of genes in any one category; and (3) the most significantly scoring 10% of genes in any two categories. Subsequently, this set of RNA binding genes was searched for degree of deletion or amplification in TCGA high-grade serous ovarian carcinoma (HGS-OvCa) dataset. Only those with deletion of more than 5% were selected for further functional validation.

### Ovarian cancer expression and survival analysis in datasets

The Oncomine database was searched to compare the expression levels of different genes in ovarian cancer datasets. Kaplan-Meier survival analyses for disease outcomes in the Australian Ovarian Cancer Study (AOCS) dataset (GSE9891, *n* = 285) were conducted using the online database (http://www.kmplot.com/). *P* values were calculated with log-rank (Mantel–Cox) test. Patients were stratified into “low” and “high” expression based on autoselect best cutoff in the database. The CSIOVDB database was also queried to validate the expression of different genes and their correlation with clinical staging, pathological differentiation degree, and clinical outcome of ovarian cancer patients.

### Reagents, cell culture, and cell transfection

α-Amanitin, DRB, and propidium iodide were purchased from Sigma. The concentration of α-amanitin used was 10 μg/mL and the final concentration of DRB used reached 100 μM. Antibodies used are as follows: SORBS2 (Abcam, ab73444), cleaved caspase 3 (Abcam, ab13847), WFDC1 (Abcam, ab126846, for western blotting analysis), WFDC1 (Sigma, HPA031411, for IHC analysis), IL-17D (Abcam, ab77185), Ki-67(Abcam, ab15580), Flag (Sigma, F3165), β-actin (Abcam, ab8226), HLA-DR (human; BD Bioscience, 556,643), CD14 (human; BD Bioscience, 555,397), CD206 (human; BioLegend, 321,121), CD45 (murine; BioLegend, 103,127), CD11b (murine; BioLegend, 101,230), GR-1 (murine; BioLegend, 108,416), CD206 (murine; BioLegend, 141,703).

All cell lines were maintained at 37 °C and 5% CO_2_. The human ovarian cancer cell lines SKOV3, CAOV3, COV434, COV644, COV362, and COV504 were cultured in Dulbecco’s modified Eagle medium supplemented with 10% fetal bovine serum (FBS), 2 mM L-glutamine, 1 mM sodium pyruvate, and 100 U/ml penicillin-streptomycin (both from Gibco-BRL, Grand Island, NY, USA). The human ovarian cancer cell lines A2780S, OVSAHO, OVTOKO, OV56, COLO-720E, OVISE, OV90, KURAMOCHI, OVCAR4, and OVCAR3 were cultured in RPMI-1640 containing 10% FBS and 100 U/ml penicillin-streptomycin (both from Gibco-BRL, Grand Island, NY, USA). The mouse ovarian cancer cell line ID-8 was cultured in Dulbecco’s modified Eagle medium supplemented with 10% FBS, 2 mM L-glutamine, 1 mM sodium pyruvate, and 100 U/ml penicillin-streptomycin (both from Gibco-BRL, Grand Island, NY, USA). Cell lines received in 2014 were tested for authenticity in 2016 using short tandem repeat (STR) genotyping.

Based upon the shRNA design principle, oligonucleotide sequences of SORBS2 and respective corresponding nontargeting negative control (NC) shRNAs were designed by OBiO Technology (Shanghai, China), which are as follows: sh1-SORBS2, forward, 5′-CGCTAACATCTGTGAAGAGcTCAAGAGACTCTTCACAGATGTTAGCG-3′, reverse, 5′-CGCTAACATCTGTGAAGAGTCTCTTGAGCTCTTCACAGATGTTAGCG-3′; sh2-SORBS2, forward, 5′-TGCAAAGTTCTCCAAACCTCTCAAGAGAAGGTTTGGAGAACTTTGCA-3′, reverse, 5′-TGCAAAGTTCTCCAAACCTTCTCTTGAgAGGTTTGGAGAACTTTGCA-3′. They were separately transfected into cells using Lipofectamine 3000 (Invitrogen) according to the manufacturer’s instructions. siRNA oligonucleotides with specificity for BTF3, SORBS2, CIRBP, and MEX3D and respective corresponding nontargeting control siRNAs were obtained from GenePharma. They were separately transfected into cells using Lipofectamine RNAiMAX (Invitrogen) according to the manufacturer’s instructions. The full-length human Flag-tagged SORBS2 plasmid, IL-17D plasmid, WFDC1 plasmid, and the three SORBS2 mutant plasmids were purchased from Gene Copoeia (Guangzhou, China). The plasmids were designed based on the cDNA sequence of SORBS2 (GenBank™ accession number NM_021069.4), IL-17D (GenBank™ accession number NM_138284.1), and WFDC1 (GenBank™ accession number NM_021197.3). They were separately transfected into cells using Lipofectamine 3000 (Invitrogen) according to the manufacturer’s instructions.

### Patients and specimens

De novo serous ovarian cancer patients and normal ovarian surface tissue specimens as controls were collected from West China Second Hospital, Sichuan University. The ascites samples of ovarian cancer patients were also obtained from West China Second Hospital, Sichuan University. All of these samples were examined by experienced pathologists who confirmed the diagnosis of disease samples.

### Immunohistochemistry

Paraffin-embedded normal ovary and ovarian cancer specimens from West China Second Hospital, Sichuan University were used for immunohistochemistry. Immunohistochemistry was performed utilizing primary antibodies listed in Additional file 6: Table S5 as previously described [34]. Ten random images per section were captured, and immunohistochemical staining positivity was determined by calculating the percentage of positive cells and immunostain intensity using Image-Pro Plus version 6.0 (Media Cybernetics, Baltimore, MD). All slides were evaluated by two independent pathologists in a double-blinded manner. Any discrepancy between the two evaluators was resolved by re-evaluation and open deliberation until agreement was reached.

### Immunoblotting

For immunoblotting, the whole cell lysates were prepared as described previously [35]. Detailed information on the primary antibodies used in immunoblotting analysis are listed in Additional file 6: Table S5. The signals were quantified by QuantityOne software (Bio-Rad) and β-actin was used as internal control.

### An orthotopic intrabursal injection model of ovarian cancer in mice

Animal studies were reviewed and approved by the Institutional Ethics Committee of Sichuan University. Female athymic BALB/c nude mice (6–8 weeks old, 18–20 g each) were used to assess the peritoneal metastasis of human ovarian cancer cell lines. Immuno-proficient female C57BL/6 mice (6–8 weeks old, 18–20 g each) were used to assess the peritoneal metastasis of the mouse ID-8 ovarian cancer cell line. An orthotopic model generated by intrabursal injection of ovarian cancer cell lines in mice was conducted as previously described to assess the peritoneal metastasis of ovarian cancer [35]. The number of metastatic nodules were counted and ascites volumes were measured at sacrifice.

### Quantitative reverse transcription PCR

The mRNA level of each gene was measured via qRT-PCR. Moreover, the change in 3′ UTR lengths of target genes was also examined via qRT-PCR for increasingly distal regions of target gene transcript 3′ UTRs relative to the level of the coding sequence of each of the genes. RNA was isolated using a total RNA isolation kit, including an on-column DNase treatment (Norgen). cDNA synthesis was carried out using the SuperScript III reverse transcriptase kit using a mixture of oligodT and random hexamers for priming (Life Technologies). qRT–PCR was conducted with Fast SYBR Green master mix (Applied Biosystems), and fluorescence was monitored using a 7900HT Fast real-time instrument (Applied Biosystems). Data were analyzed using the ΔΔCt method. Endogenous control transcripts were used for normalization. Statistical significance was determined using a one-tailed Student’s *t*-test. The sequences of the primers used for all qRT–PCR assays are listed in Additional file 7: Table S6.

### Transwell invasion assay and wound healing assay

Transwell 24-well chambers (Corning) were used for in vitro cell migration assays as described previously [36]. Ten contiguous fields of each sample were examined to obtain a representative number of cells that had migrated across the membrane. Wounds were scratched in confluent cells using a pipette tip, and the cells were then rinsed with medium to remove free-floating cells and debris. Serum-free medium was subsequently added, and culture plates were incubated at 37 °C for 2 days. Wound healing was observed at 0 and 48 h within the scrape line, and representative scrape lines for each cell line were photographed.

### Transcriptome sequencing

Whole-transcriptome sequencing libraries were constructed as described previously [37]. The libraries were sequenced on the Illumina HiSeq platform (Novogen, China). Reads were first trimmed to remove linker sequences and low-quality bases using Cutadapt (version 1.2.1). TopHat2 (version 2.0.8) was then used to map the reads to the human transcriptome (RefSeq transcriptome index hg19). Cufflinks (version 2.0.2) was then used to estimate RPKM (reads per kilobase per million mapped reads) values and compare the two samples. For each group, two replicates were used for the analysis.

### RIP sequencing

Cell lysates of A2780s ovarian cancer cells transiently transfected with Flag-SORBS2 plasmid were used for the RNA immunoprecipitation assay as described previously [38]. An antibody raised against Flag was conjugated to protein A Dynabeads (Life Technologies) and used to immunoprecipitate endogenous SORBS2–RNA complexes, with serum (IGG) as the control group. RNA was extracted using Trizol following the manufacturer’s instructions (Invitrogen). rRNAs were removed from the immunoprecipitated RNA and input RNA samples by using Ribo-Zero™ rRNA Removal Kit (Illumina, San Diego, CA, USA). RNA libraries were constructed by using rRNA-depleted RNAs with TruSeq Stranded Total RNA Library Prep Kit (Illumina, San Diego, CA, USA) according to the manufacturer’s instructions. Libraries were controlled for quality and quantified using the BioAnalyzer 2100 system (Agilent Technologies, Inc., USA). Libraries (10 pM) were denatured as single-stranded DNA molecules, captured on Illumina flow cells, amplified in situ as clusters and finally sequenced for 150 cycles on an Illumina HiSeq Sequencer according to the manufacturer’s instructions at Cloudseq, Shanghai in China.

### Transcript stability assays

For the α-amanitin RNA sequencing data, A2780s SORBS2-knockdown or control cells were treated with 10 μg/mL α-amanitin (Sigma). Nine hours after α-amanitin treatment, RNA was isolated from the cells using a total RNA isolation kit and subsequently subjected to RNA sequencing. We compared and identified the different mRNAs in shControl and sh1-SORBS2 samples at 0 and 9 h after α-amanitin treatment. The differences between the sh1-SORBS2/shControl log fold changes were used as a measure of stability. For validation of the RNA sequencing results, relative transcript levels were assessed by qRT-PCR, and 18S was used as an endogenous normalization control. Statistical significance was determined using a one-tailed Student’s *t*-test. Cells were seeded at 2 × 10^5^ per well in six-well plates. Eighteen hours after seeding, DRB (Sigma) was added to the cells to a final concentration of 100 μM. RNA was isolated at 0, 2, 4, 6, and 8 h after DRB addition using a total RNA isolation kit with on-column DNase treatment (Norgen). Relative levels of the transcripts of interest were assessed by qRT-PCR, using 18S as the endogenous control. Half-life calculations were done using the formula t1/2 = ln 2/kdecay, where the decay constant was determined by plotting the data on a semilog scale and using nonlinear regression to find the best fit line (Graphpad Prism version 6).

### TCGA expression analysis

RNA-seq V2 level 3 data were downloaded for 354 HGS-OvCa samples from TCGA data portal using TCGA biolinks package. The count values were transformed to log2 counts per million using the voom function from the limma R package. High and low composite groups for SORBS2 (log fold change 0.75), SORBS2 and WFDC1(log fold change 0.75), and SORBS2 and IL17D (log fold change 0.75) were defined using overlapping samples for both genes of a pair in the top and bottom 30% of expression values. Differentially expressed genes were determined using limma 0.75 fold change in expression between the combined high and low groups and were filtered to a curated list of immune factors (Additional file 8: Table S7) for visualization on heatmaps.

GSEA analysis was performed with the enrichment statistic equal to weighted, and the metric for ranking genes equal to signal-to-noise. Ovarian cancer gene expression data were also obtained from TCGA dataset. False discovery rate (FDR) q values of < 0.25 or nominal (NOM) *P* values of < 0.05 were considered to be significant.

### Flow cytometry

For flow cytometry-based apoptosis analysis, we lysed the tumor tissues in each group, stained the cells with the FITC Annexin V Apoptosis Detection Kit with PI (BioLegend, 640,914), and then analyzed them by the flow cytometry assay. For the cell cycle analysis, cells in each group were stained by propidium iodide (Sigma, P4170) and then analyzed by the flow cytometry assay.

Human CD14+ cells were isolated from healthy donor peripheral blood mononuclear cells (PBMCs) using magnetic CD14+ microbeads and incubated with conditioned media from A2780s ovarian cancer cells transfected with shCtrl, SORBS2-depleted A2780s cells, WFDC1-overexpressing and SORBS2-depleted A2780s cells, and IL-17D-overexpressing and SORBS2-depleted A2780s cells. After 48 h, treated myeloid cells were stained with fluorochrome-labeled antibodies against HLA-DR, CD14, and CD206 and analyzed by flow cytometry. Mouse metastatic nodules were cut into small pieces followed by passage through a 40-μM nylon filter (BD). Red blood cells were lysed for 5 min at room temperature (RT) in RBC lysis buffer (BioLegend). Cells were then labeled with fixable viability dye eFluor780 (eBioscience) for 30 min in the dark at RT, followed by Fc block (BD Biosciences). Antibodies used were against CD45, CD11b, GR-1, and CD206. Detailed information is shown in Additional file 6: Table S5.

### Statistics

The data are presented as the means ± SD of three independent experiments unless otherwise indicated. GraphPad Prism (GraphPad Software Inc., La Jolla, CA) was applied for data analysis with all data assessed for normal distribution and equal variance. The correlation analysis was analyzed using a linear regression analysis. Comparisons between two groups were performed with Student’s *t*-test, and differences among multiple groups were evaluated by one-way analysis of variance. The survival of different treatment groups were analyzed by Kaplan-Meier analysis. Statistical significance is considered as *P* < 0.05.

## Additional files

Additional file 1: **Table S1.** A high-priority list of 145 RBP genes associated with ovarian cancer aggressiveness. (XLSX 12 kb)

Additional file 2: **Figures S1**–**S14.** with figure legends. (PDF 1843 kb)

Additional file 3: **Table S2.** The relative SORBS2 expression in ovarian cancer cell lines of different molecular subtypes. (XLSX 11 kb)

Additional file 4: **Table S3.** Gene names for the transcripts potentially bound and stabilized by SORBS2 in ovarian cancer. (XLSX 15 kb)

Additional file 5: **Table S4.** Enrichment of gene ontology biological processes and KEGG pathways for the transcripts potentially bound and stabilized by SORBS2 in ovarian cancer. (XLSX 12 kb)

Additional file 6: **Table S5.** Antibodies used in this study. (XLSX 11 kb)

Additional file 7: **Table S6.** Primer sequences for selected genes. (XLSX 12 kb)

Additional file 8: **Table S7.** Reported immune cell markers and cytokines. (XLSX 11 kb)

## References

[1] Bowtell DD, Bohm S, Ahmed AA, Aspuria PJ, Bast RC, Beral V, Berek JS, Birrer MJ, Blagden S, Bookman MA (2015). Rethinking ovarian cancer II: reducing mortality from high-grade serous ovarian cancer. Nat Rev Cancer.

[2] Karnezis AN, Cho KR, Gilks CB, Pearce CL, Huntsman DG (2017). The disparate origins of ovarian cancers: pathogenesis and prevention strategies. Nat Rev Cancer.

[3] Matulonis UA, Sood AK, Fallowfield L, Howitt BE, Sehouli J, Karlan BY (2016). Ovarian cancer. Nat Rev Dis Primers.

[4] Luo Z, Wang Q, Lau WB, Lau B, Xu L, Zhao L, Yang H, Feng M, Xuan Y, Yang Y (2016). Tumor microenvironment: The culprit for ovarian cancer metastasis?. Cancer Lett.

[5] Kitamura T, Qian BZ, Pollard JW (2015). Immune cell promotion of metastasis. Nat Rev Immunol.

[6] Dunn GP, Koebel CM, Schreiber RD (2006). Interferons, immunity and cancer immunoediting. Nat Rev Immunol.

[7] Jimenez-Sanchez A, Memon D, Pourpe S, Veeraraghavan H, Li Y, Vargas HA, Gill MB, Park KJ, Zivanovic O, Konner J (2017). Heterogeneous tumor-immune microenvironments among differentially growing metastases in an ovarian cancer patient. Cell.

[8] Montfort A, Pearce O, Maniati E, Vincent BG, Bixby L, Bohm S, Dowe T, Wilkes EH, Chakravarty P, Thompson R (2017). A strong B-cell response is part of the immune landscape in human high-grade serous ovarian metastases. Clin Cancer Res.

[9] Bowen NJ, Walker LD, Matyunina LV, Logani S, Totten KA, Benigno BB, McDonald JF (2009). Gene expression profiling supports the hypothesis that human ovarian surface epithelia are multipotent and capable of serving as ovarian cancer initiating cells. BMC Med Genet.

[10] Brodsky AS, Fischer A, Miller DH, Vang S, MacLaughlan S, Wu HT, Yu J, Steinhoff M, Collins C, Smith PJ (2014). Expression profiling of primary and metastatic ovarian tumors reveals differences indicative of aggressive disease. PLoS One.

[11] Condello S, Morgan CA, Nagdas S, Cao L, Turek J, Hurley TD, Matei D (2015). beta-catenin-regulated ALDH1A1 is a target in ovarian cancer spheroids. Oncogene.

[12] Tan TZ, Yang H, Ye J, Low J, Choolani M, Tan DS, Thiery JP, Huang RY (2015). CSIOVDB: a microarray gene expression database of epithelial ovarian cancer subtype. Oncotarget.

[13] Tothill RW, Tinker AV, George J, Brown R, Fox SB, Lade S, Johnson DS, Trivett MK, Etemadmoghadam D, Locandro B (2008). Novel molecular subtypes of serous and endometrioid ovarian cancer linked to clinical outcome. Clin Cancer Res.

[14] Integrated genomic analyses of ovarian carcinoma. Nature 2011, 474:609–615. https://cancergenome.nih.gov/. 10.1038/nature10166 PMC3163504 21720365

[15] Fish L, Pencheva N, Goodarzi H, Tran H, Yoshida M, Tavazoie SF (2016). Muscleblind-like 1 suppresses breast cancer metastatic colonization and stabilizes metastasis suppressor transcripts. Genes Dev.

[16] Coleman RL (2016). Ovarian cancer in 2015: Insights into strategies for optimizing ovarian cancer care. Nat Rev Clin Oncol.

[17] Calabretta S, Richard S (2015). Emerging roles of disordered sequences in RNA-binding proteins. Trends Biochem Sci.

[18] Anekal PV, Yong J, Manser E (2015). Arg kinase-binding protein 2 (ArgBP2) interaction with alpha-actinin and actin stress fibers inhibits cell migration. J Biol Chem.

[19] Zhang Q, Gao X, Li C, Feliciano C, Wang D, Zhou D, Mei Y, Monteiro P, Anand M, Itohara S (2016). Impaired dendritic development and memory in Sorbs2 knock-out mice. J Neurosci.

[20] Tong Y, Li Y, Gu H, Wang C, Liu F, Shao Y, Li J, Cao L, Li F (2015). Microchidia protein 2, MORC2, downregulates the cytoskeleton adapter protein, ArgBP2, via histone methylation in gastric cancer cells. Biochem Biophys Res Commun.

[21] Wang H, Bei Y, Shen S, Huang P, Shi J, Zhang J, Sun Q, Chen Y, Yang Y, Xu T (2016). miR-21-3p controls sepsis-associated cardiac dysfunction via regulating SORBS2. J Mol Cell Cardiol.

[22] Robin JD, Ludlow AT, Batten K, Gaillard MC, Stadler G, Magdinier F, Wright WE, Shay JW (2015). SORBS2 transcription is activated by telomere position effect-over long distance upon telomere shortening in muscle cells from patients with facioscapulohumeral dystrophy. Genome Res.

[23] Ressler SJ, Rowley DR (2011). The WFDC1 gene: role in wound response and tissue homoeostasis. Biochem Soc Trans.

[24] Madar S, Brosh R, Buganim Y, Ezra O, Goldstein I, Solomon H, Kogan I, Goldfinger N, Klocker H, Rotter V (2009). Modulated expression of WFDC1 during carcinogenesis and cellular senescence. Carcinogenesis.

[25] Starnes T, Broxmeyer HE, Robertson MJ, Hromas R (2002). Cutting edge: IL-17D, a novel member of the IL-17 family, stimulates cytokine production and inhibits hemopoiesis. J Immunol.

[26] O'Sullivan T, Saddawi-Konefka R, Gross E, Tran M, Mayfield SP, Ikeda H, Bui JD (2014). Interleukin-17D mediates tumor rejection through recruitment of natural killer cells. Cell Rep.

[27] Ressler SJ, Dang TD, Wu SM, Tse DY, Gilbert BE, Vyakarnam A, Yang F, Schauer IG, Barron DA, Rowley DR (2014). WFDC1 is a key modulator of inflammatory and wound repair responses. Am J Pathol.

[28] Saddawi-Konefka R, Seelige R, Gross ET, Levy E, Searles SC, Washington A, Santosa EK, Liu B, O'Sullivan TE, Harismendy O, Bui JD (2016). Nrf2 induces IL-17D to mediate tumor and virus surveillance. Cell Rep.

[29] Castello A, Fischer B, Eichelbaum K, Horos R, Beckmann BM, Strein C, Davey NE, Humphreys DT, Preiss T, Steinmetz LM (2012). Insights into RNA biology from an atlas of mammalian mRNA-binding proteins. Cell.

[30] Baltz AG, Munschauer M, Schwanhausser B, Vasile A, Murakawa Y, Schueler M, Youngs N, Penfold-Brown D, Drew K, Milek M (2012). The mRNA-bound proteome and its global occupancy profile on protein-coding transcripts. Mol Cell.

[31] Ray D, Kazan H, Cook KB, Weirauch MT, Najafabadi HS, Li X, Gueroussov S, Albu M, Zheng H, Yang A (2013). A compendium of RNA-binding motifs for decoding gene regulation. Nature.

[32] Kwon SC, Yi H, Eichelbaum K, Fohr S, Fischer B, You KT, Castello A, Krijgsveld J, Hentze MW, Kim VN (2013). The RNA-binding protein repertoire of embryonic stem cells. Nat Struct Mol Biol.

[33] Cook KB, Kazan H, Zuberi K, Morris Q, Hughes TR (2011). RBPDB: a database of RNA-binding specificities. Nucleic Acids Res.

[34] Zhao L, Ji G, Le X, Wang C, Xu L, Feng M, Zhang Y, Yang H, Xuan Y, Yang Y (2017). Long noncoding RNA LINC00092 acts in cancer-associated fibroblasts to drive glycolysis and progression of ovarian cancer. Cancer Res.

[35] Zhao L, Ji G, Le X, Luo Z, Wang C, Feng M, Xu L, Zhang Y, Lau WB, Lau B (2017). An integrated analysis identifies STAT4 as a key regulator of ovarian cancer metastasis. Oncogene.

[36] Zhao L, Zhou S, Zou L, Zhao X (2013). The expression and functionality of stromal caveolin 1 in human adenomyosis. Hum Reprod.

[37] Yu Y, Chen Y, Kim B, Wang H, Zhao C, He X, Liu L, Liu W, Wu LM, Mao M (2013). Olig2 targets chromatin remodelers to enhancers to initiate oligodendrocyte differentiation. Cell.

[38] Cannell IG, Merrick KA, Morandell S, Zhu CQ, Braun CJ, Grant RA, Cameron ER, Tsao MS, Hemann MT, Yaffe MB (2015). A pleiotropic RNA-binding protein controls distinct cell cycle checkpoints to drive resistance of p53-defective tumors to chemotherapy. Cancer Cell.

[39] Zhao L, Wang W, Huang S, Yang Z, Xu L, Yang Q, Zhou X, Wang J, Shen Q, Wang C, Le X, et al. The RNA binding protein SORBS2 suppresses metastatic colonization of ovarian cancer by stabilizing tumor-suppressive immunomodulatory transcripts. Genome Biology. 2018. 10.1186/s13059-018-1412-6 PMC5857099 29548303

[40] Anglesio MS, Arnold JM, George J, Tinker AV, Tothill R, Waddell N, Simms L, Locandro B, Fereday S, Traficante N (2008). Mutation of ERBB2 provides a novel alternative mechanism for the ubiquitous activation of RAS-MAPK in ovarian serous low malignant potential tumors. Mol Cancer Res.

[41] Gilks CB, Vanderhyden BC, Zhu S, van de Rijn M, Longacre TA (2005). Distinction between serous tumors of low malignant potential and serous carcinomas based on global mRNA expression profiling. Gynecol Oncol.

