# Supplementary material for: The RNA binding protein SORBS2 suppresses metastatic colonization of ovarian cancer by stabilizing tumor-suppressive immunomodulatory transcripts
Source: Genome Biol. 2018 Mar 16;19:35. doi: 10.1186/s13059-018-1412-6 (PMC5857099; doi:10.1186/s13059-018-1412-6)
Supplement: Supplementary file 2 — Figures S1–S14. with figure legends. (PDF 1843 kb) [file 13059_2018_1412_MOESM2_ESM.pdf]

Supplementary Figures

Figure S1

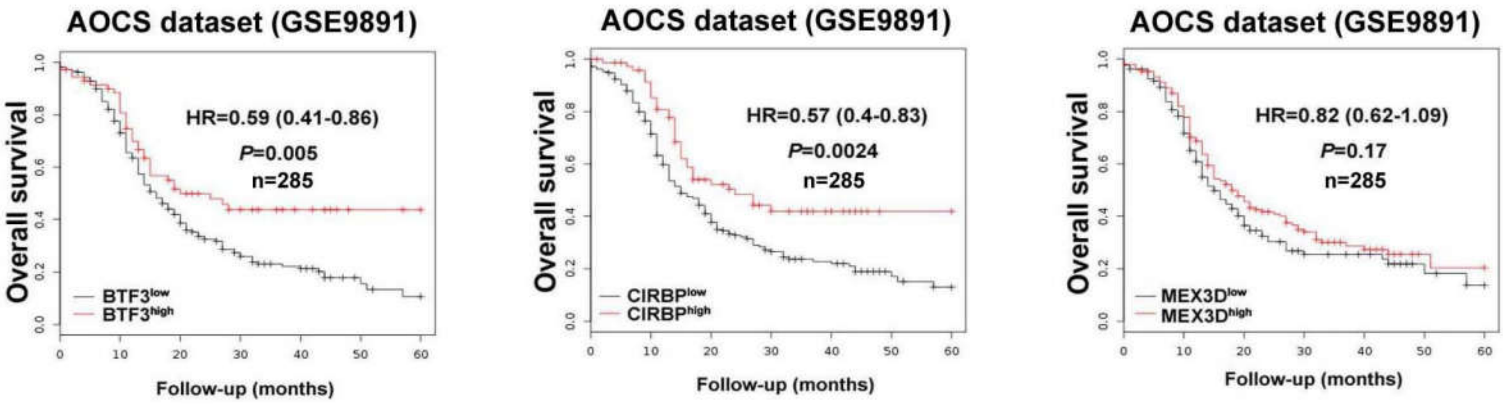

Figure S2

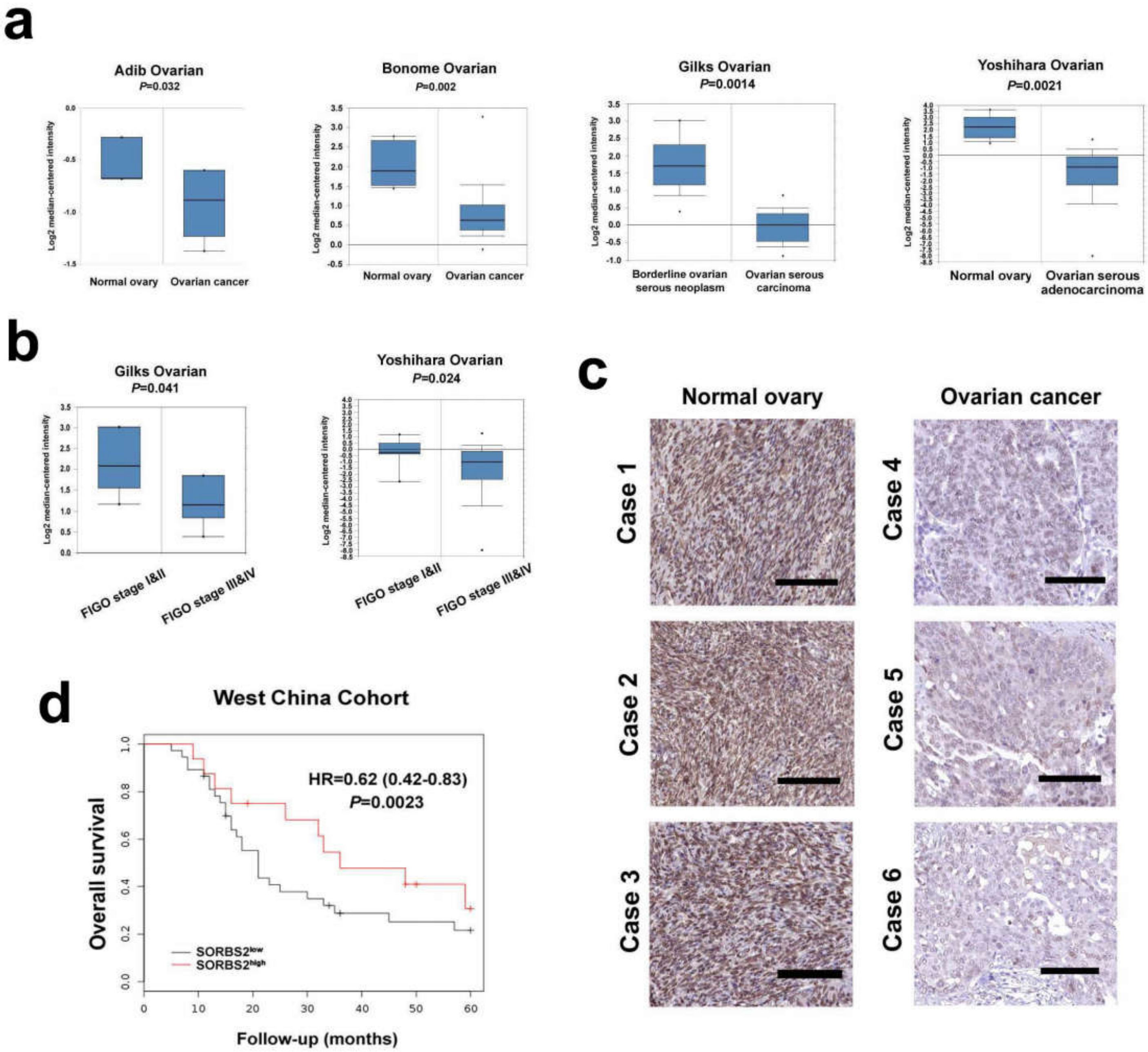
a
Adib Ovarian
P=0.032
Log2 median-centered intensity
Normal ovary
Ovarian cancer
Bonome Ovarian
P=0.002
Log2 median-centered intensity
Normal ovary
Ovarian cancer
Gilks Ovarian
P=0.0014
Log2 median-centered intensity
Borderline ovarian serous neoplasm
Ovarian serous carcinoma
Yoshihara Ovarian
P=0.0021
Log2 median-centered intensity
Normal ovary
Ovarian serous adenocarcinoma
b
Gilks Ovarian
P=0.041
Log2 median-centered intensity
FIGO stage I&II
FIGO stage III&IV
Yoshihara Ovarian
P=0.024
Log2 median-centered intensity
FIGO stage I&II
FIGO stage III&IV
c
Normal ovary
Ovarian cancer
Case 1
Case 2
Case 3
Case 4
Case 5
Case 6
d
West China Cohort
HR=0.62 (0.42-0.83)
P=0.0023
Overall survival
SORBS2low
SORBS2high
Follow-up (months)

Figure S3

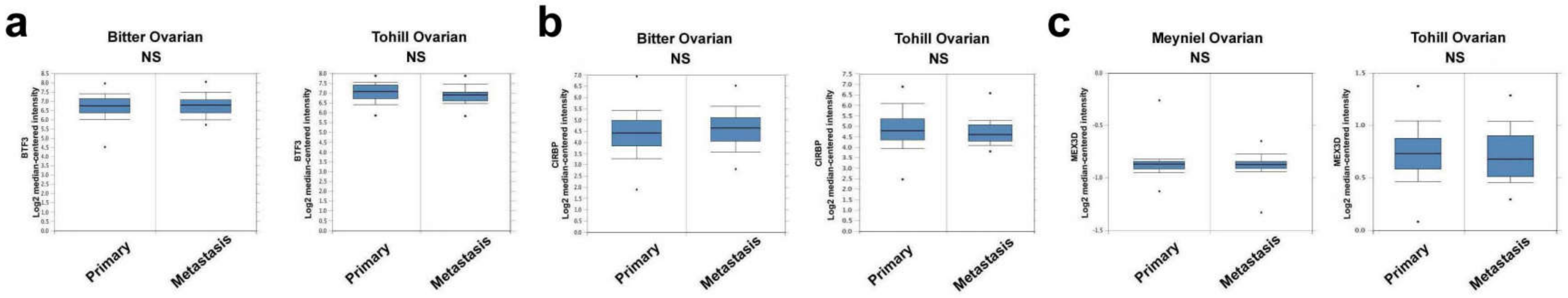
a
Bitter Ovarian
NS
BTF3
Log2 median-centered intensity
Primary
Metastasis
Tohill Ovarian
NS
BTF3
Log2 median-centered intensity
Primary
Metastasis
b
Bitter Ovarian
NS
CIRBP
Log2 median-centered intensity
Primary
Metastasis
Tohill Ovarian
NS
CIRBP
Log2 median-centered intensity
Primary
Metastasis
c
Meyniel Ovarian
NS
MEX3D
Log2 median-centered intensity
Primary
Metastasis
Tohill Ovarian
NS
MEX3D
Log2 median-centered intensity
Primary
Metastasis

Figure S4

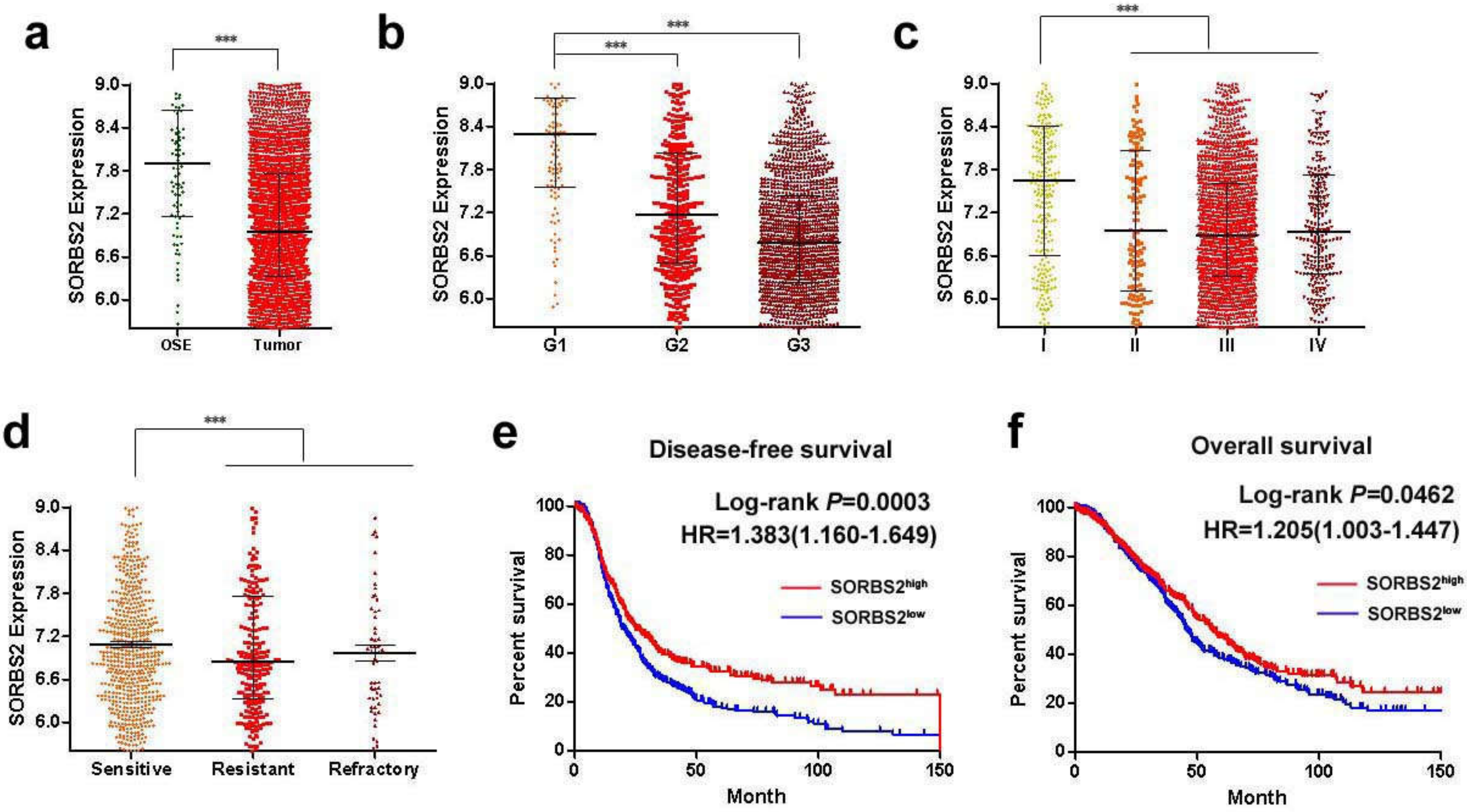

a
***
SORBS2 Expression
9.0
8.4
7.8
7.2
6.6
6.0
OSE
Tumor
b
***
***
G1
G2
G3
c
I
II
III
IV
d
Sensitive
Resistant
Refractory
e
Disease-free survival
Log-rank P=0.0003
HR=1.383(1.160-1.649)
SORBS2high
SORBS2low
Percent survival
Month
f
Overall survival
Log-rank P=0.0462
HR=1.205(1.003-1.447)

Figure S5

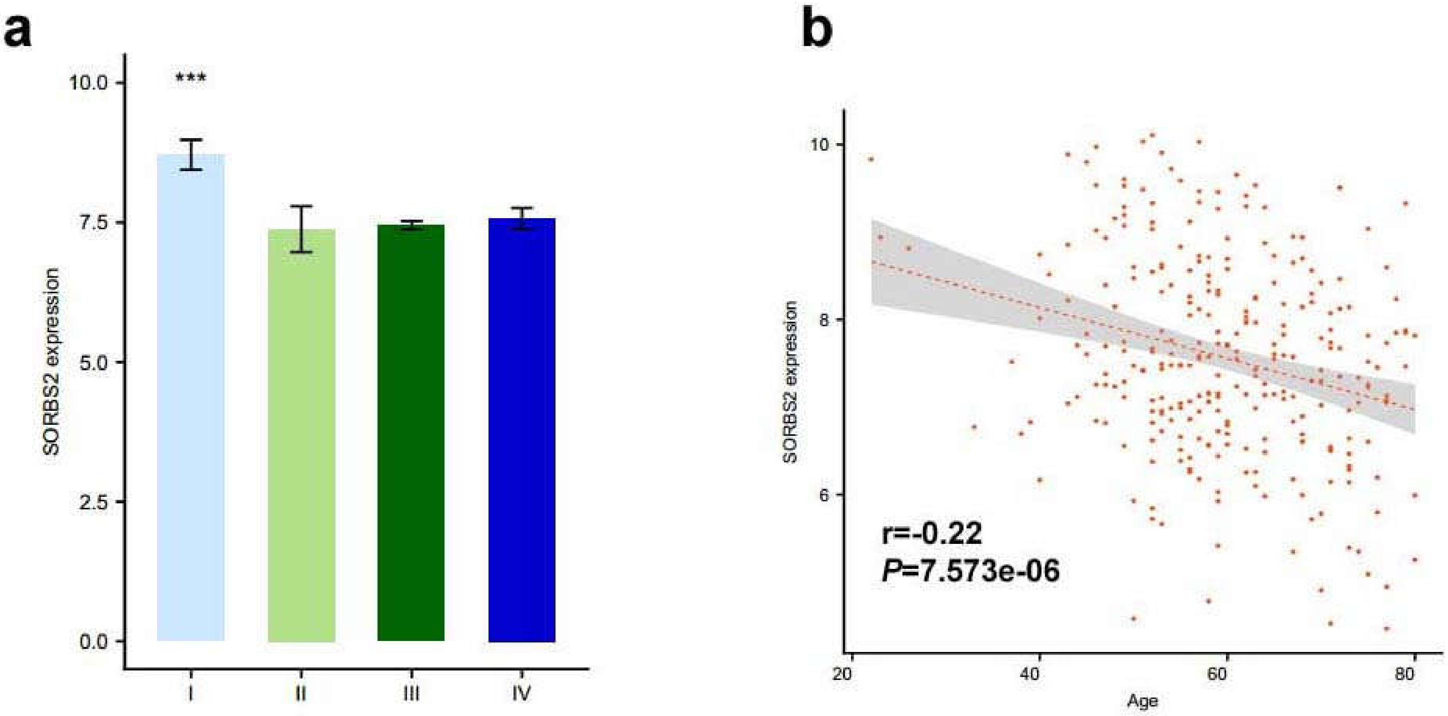
a
***
10.0
7.5
5.0
2.5
0.0
SORBS2 expression
I
II
III
IV
b
10
8
6
SORBS2 expression
r=-0.22
P=7.573e-06
20
40
60
80
Age

Figure S6

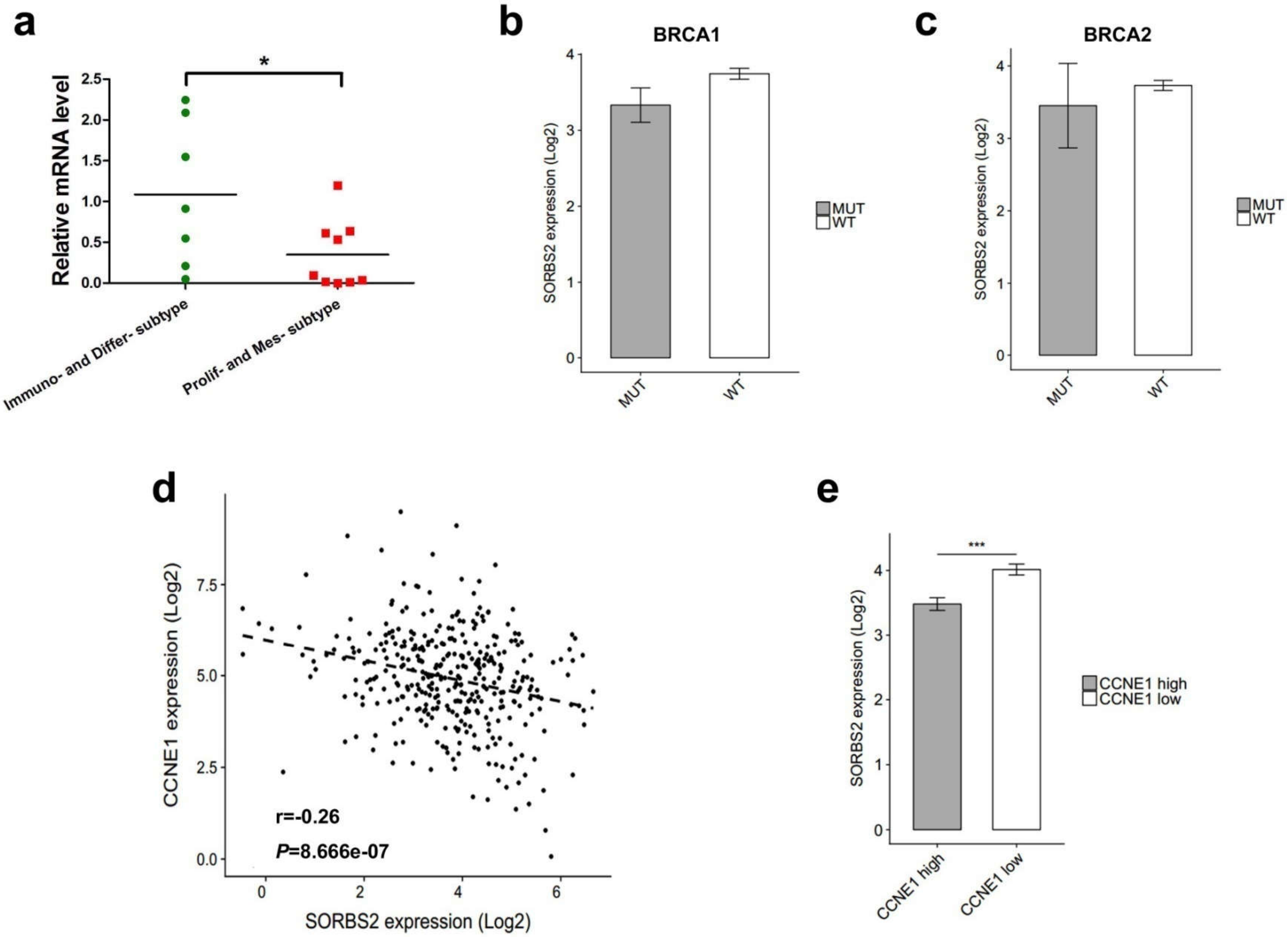
a
Relative mRNA level
2.5
2.0
1.5
1.0
0.5
0.0
*
Immuno- and Differ- subtype
Prolif- and Mes- subtype
b
BRCA1
SORBS2 expression (Log2)
MUT
WT
c
BRCA2
SORBS2 expression (Log2)
MUT
WT
d
CCNE1 expression (Log2)
7.5
5.0
2.5
0.0
0
2
4
6
SORBS2 expression (Log2)
r=-0.26
P=8.666e-07
e
SORBS2 expression (Log2)
***
CCNE1 high
CCNE1 low

Figure S7

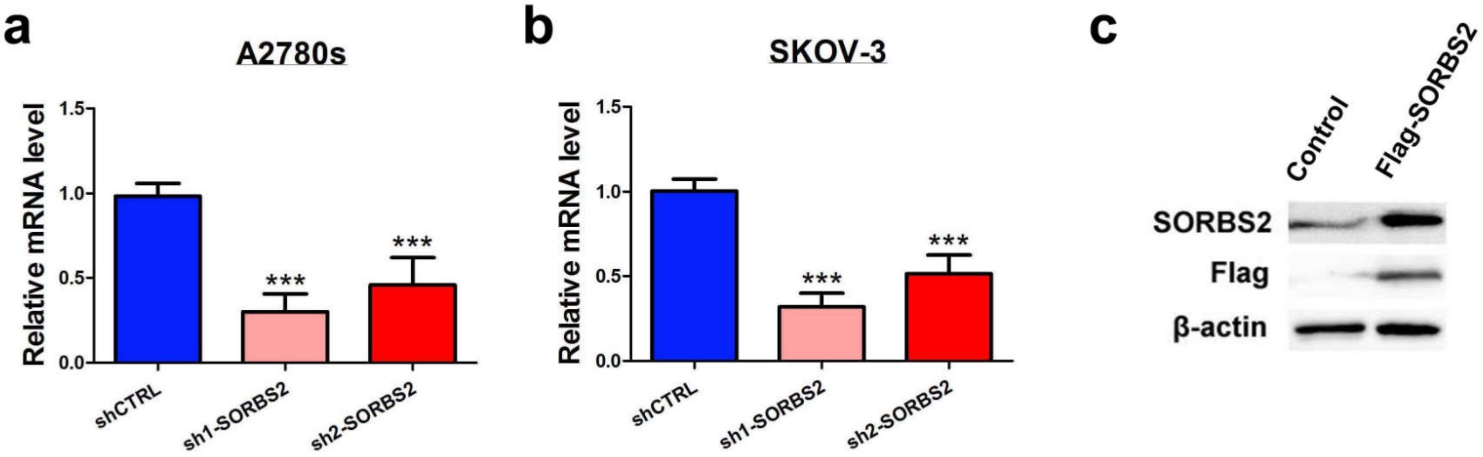
a
A2780s
Relative mRNA level
1.5
1.0
0.5
0.0
***
***
shCTRL
sh1-SORBS2
sh2-SORBS2
b
SKOV-3
Relative mRNA level
1.5
1.0
0.5
0.0
***
***
shCTRL
sh1-SORBS2
sh2-SORBS2
c
Control
Flag-SORBS2
SORBS2
Flag
β-actin

Figure S8

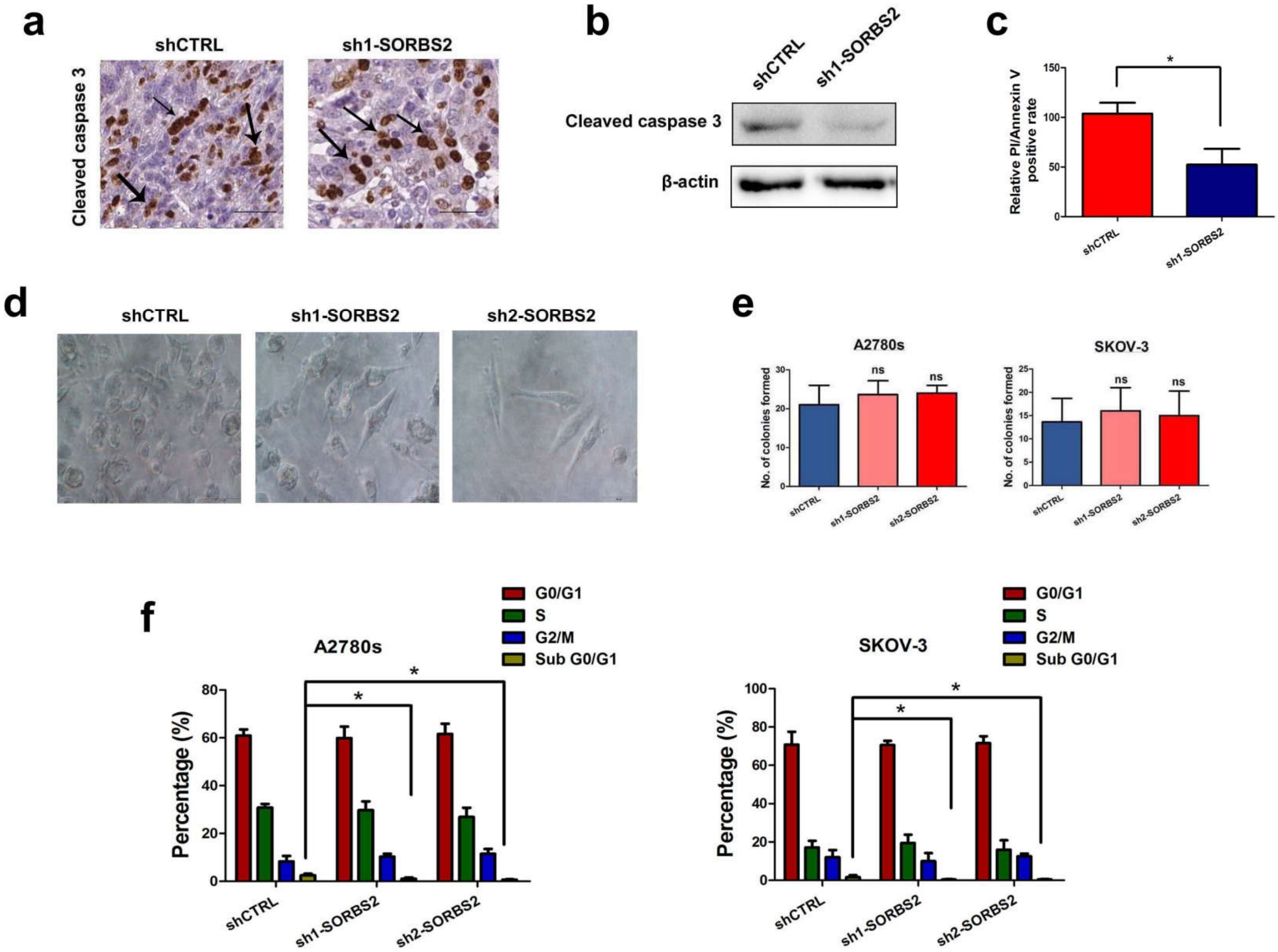
a
shCTRL
sh1-SORBS2
Cleaved caspase 3
b
shCTRL
sh1-SORBS2
Cleaved caspase 3
β-actin
c
Relative PI/Annexin V positive rate
shCTRL
sh1-SORBS2
d
shCTRL
sh1-SORBS2
sh2-SORBS2
e
A2780s
SKOV-3
No. of colonies formed
ns
shCTRL
sh1-SORBS2
sh2-SORBS2
f
A2780s
SKOV-3
G0/G1
S
G2/M
Sub G0/G1
Percentage (%)
shCTRL
sh1-SORBS2
sh2-SORBS2

Figure S9

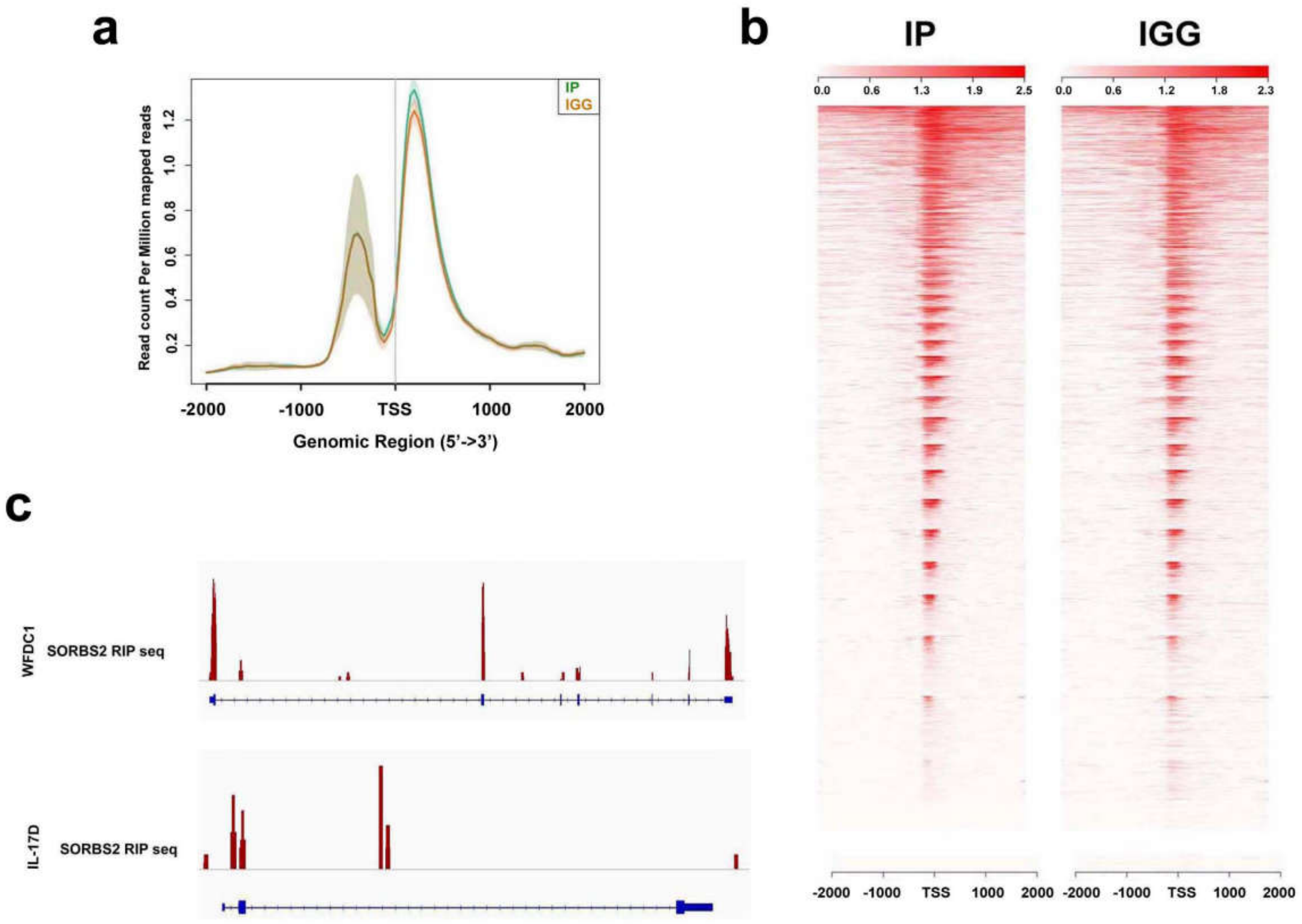
a
b
IP
IGG
Read count Per Million mapped reads
0.2
0.4
0.6
0.8
1.0
1.2
-2000
-1000
TSS
1000
2000
Genomic Region (5'->3')
0.0
0.6
1.3
1.9
2.5
1.2
1.8
2.3
c
WFDC1
SORBS2 RIP seq
IL-17D
SORBS2 RIP seq

Figure S10

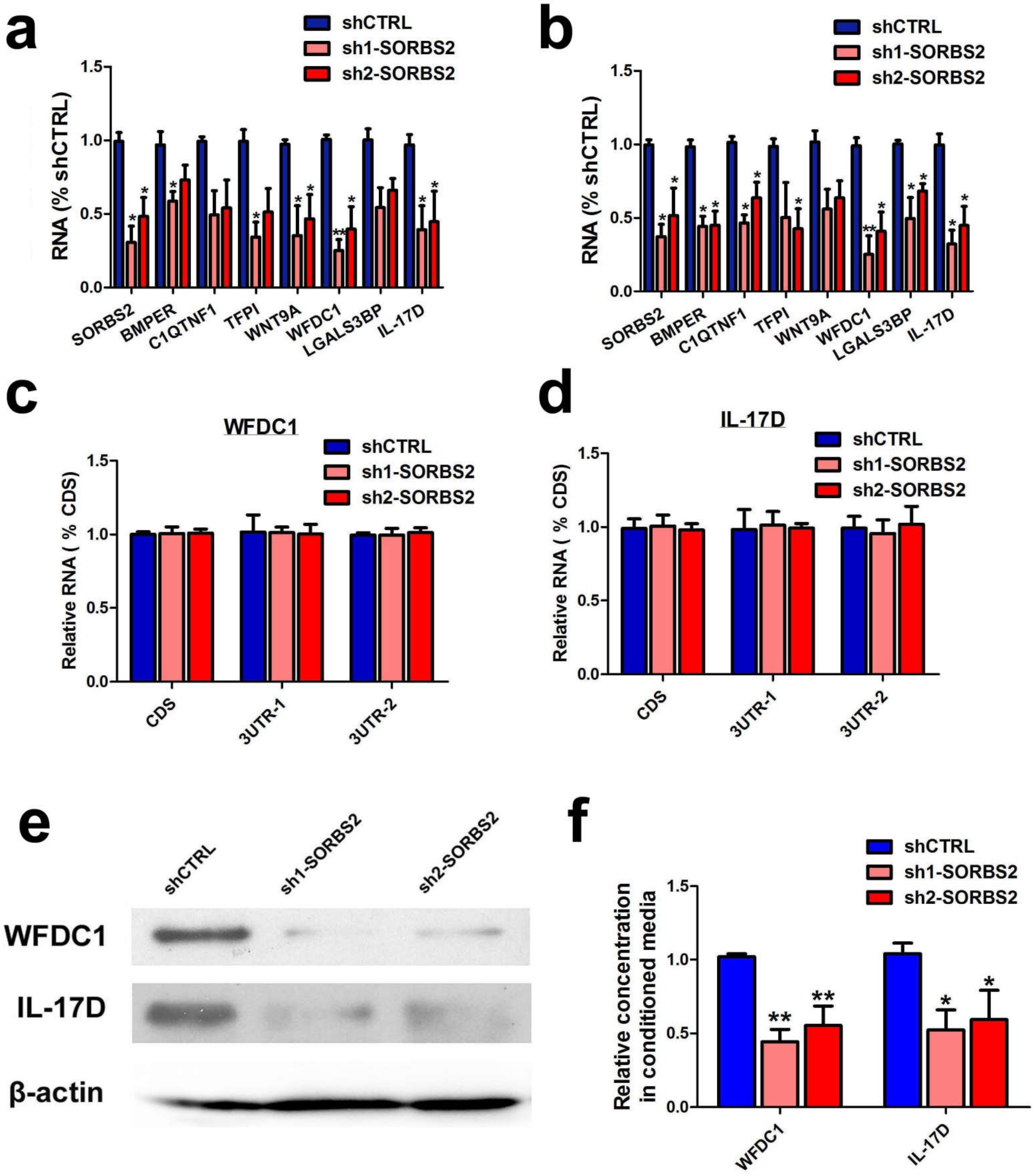
a
shCTRL
sh1-SORBS2
sh2-SORBS2
RNA (% shCTRL)
SORBS2
BMPER
C1QTNF1
TFPI
WNT9A
WFDC1
LGALS3BP
IL-17D
b
c
WFDC1
Relative RNA (% CDS)
CDS
3UTR-1
3UTR-2
d
IL-17D
e
shCTRL
sh1-SORBS2
sh2-SORBS2
WFDC1
IL-17D
β-actin
f
Relative concentration in conditioned media

Figure S11

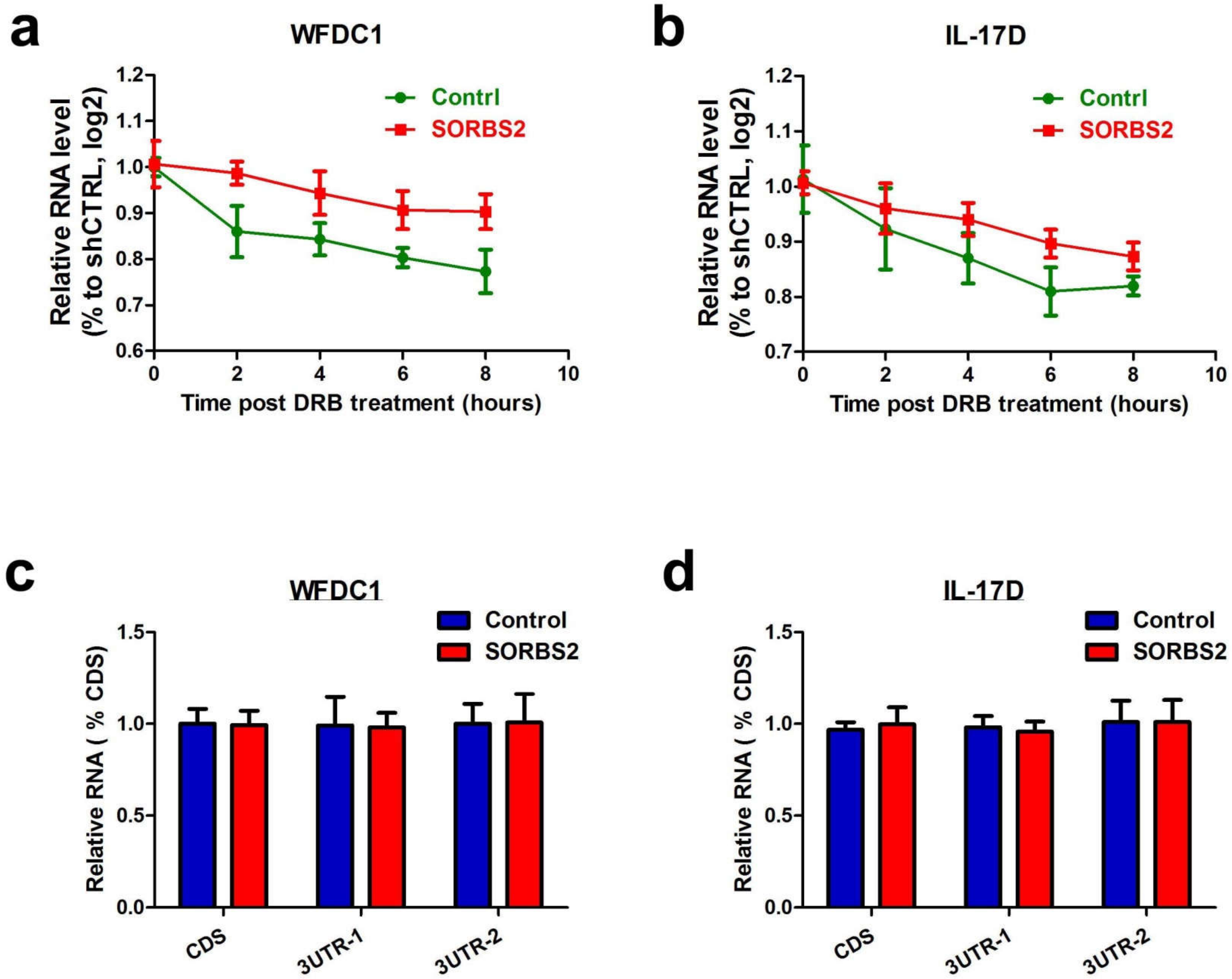
a
WFDC1
Contrl
SORBS2
Relative RNA level
(% to shCTRL, log2)
1.2
1.1
1.0
0.9
0.8
0.7
0.6
0
2
4
6
8
10
Time post DRB treatment (hours)
b
IL-17D
Contrl
SORBS2
Relative RNA level
(% to shCTRL, log2)
1.2
1.1
1.0
0.9
0.8
0.7
0
2
4
6
8
10
Time post DRB treatment (hours)
c
WFDC1
Control
SORBS2
Relative RNA (% CDS)
1.5
1.0
0.5
0.0
CDS
3UTR-1
3UTR-2
d
IL-17D
Control
SORBS2
Relative RNA (% CDS)
1.5
1.0
0.5
0.0
CDS
3UTR-1
3UTR-2

Figure S12

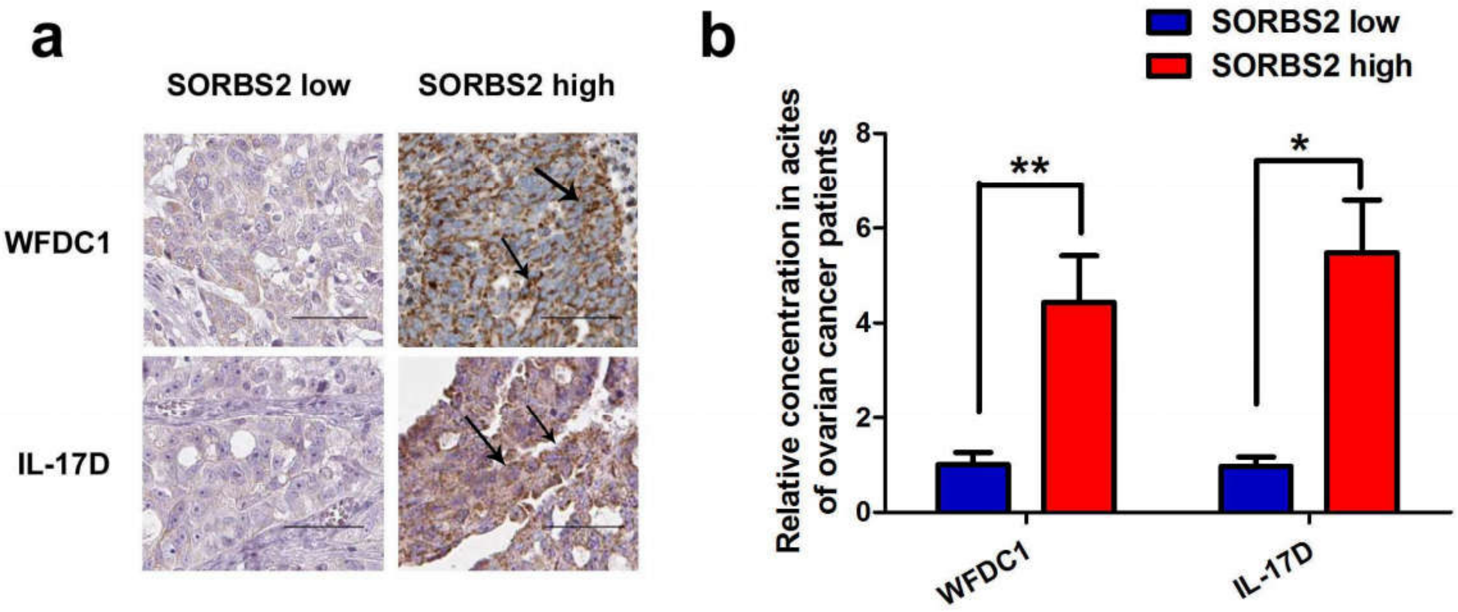
a
SORBS2 low
SORBS2 high
WFDC1
IL-17D
b
SORBS2 low
SORBS2 high
Relative concentration in acites of ovarian cancer patients
8
6
4
2
0
**
*
WFDC1
IL-17D

Figure S13

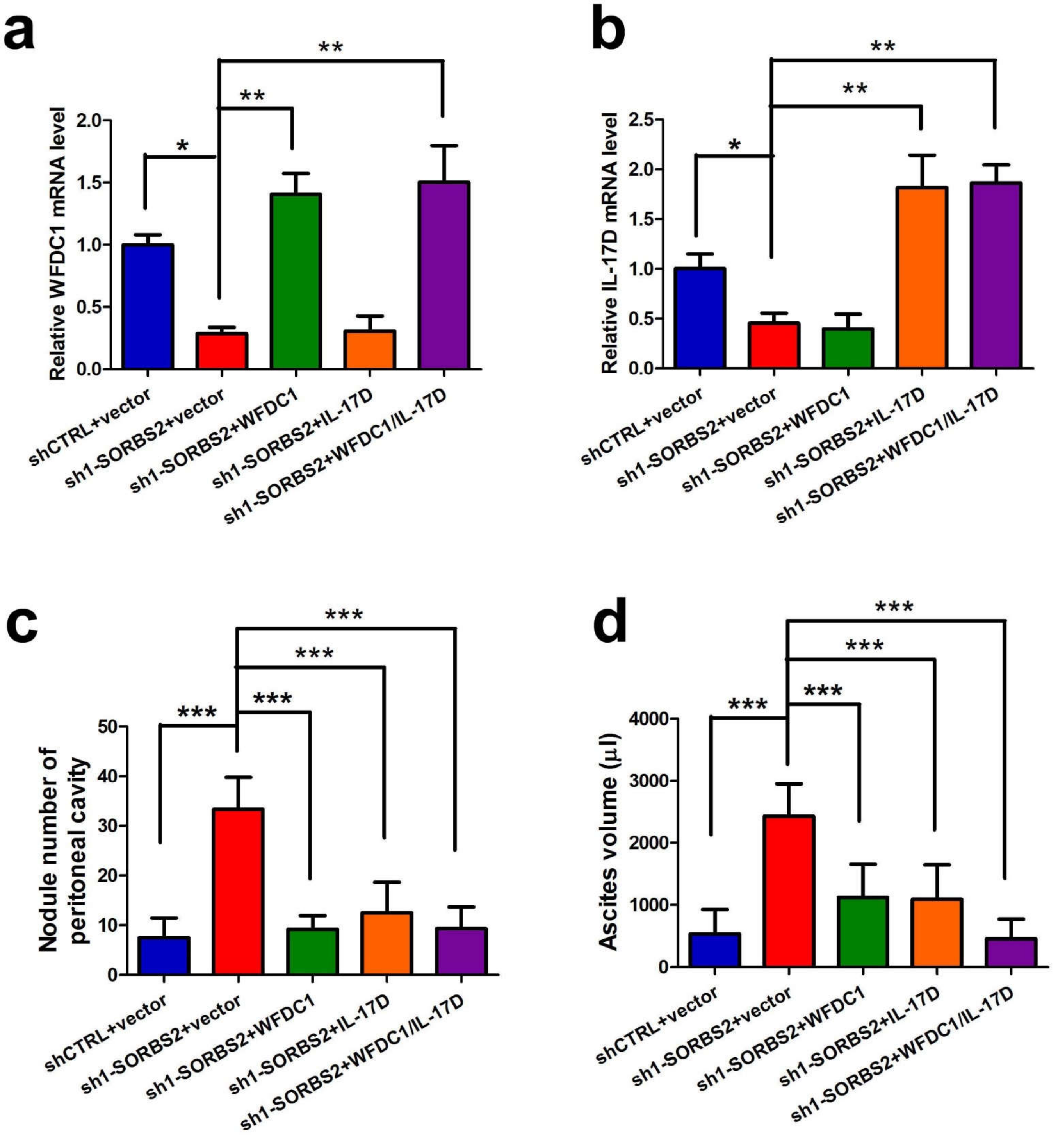
a
Relative WFDC1 mRNA level
2.0
1.5
1.0
0.5
0.0
*
**
**
shCTRL+vector
sh1-SORBS2+vector
sh1-SORBS2+WFDC1
sh1-SORBS2+IL-17D
sh1-SORBS2+WFDC1/IL-17D
b
Relative IL-17D mRNA level
2.5
2.0
1.5
1.0
0.5
0.0
*
**
**
shCTRL+vector
sh1-SORBS2+vector
sh1-SORBS2+WFDC1
sh1-SORBS2+IL-17D
sh1-SORBS2+WFDC1/IL-17D
c
Nodule number of peritoneal cavity
50
40
30
20
10
0
***
***
***
***
shCTRL+vector
sh1-SORBS2+vector
sh1-SORBS2+WFDC1
sh1-SORBS2+IL-17D
sh1-SORBS2+WFDC1/IL-17D
d
Ascites volume (µl)
4000
3000
2000
1000
0
***
***
***
***
shCTRL+vector
sh1-SORBS2+vector
sh1-SORBS2+WFDC1
sh1-SORBS2+IL-17D
sh1-SORBS2+WFDC1/IL-17D

Figure S14

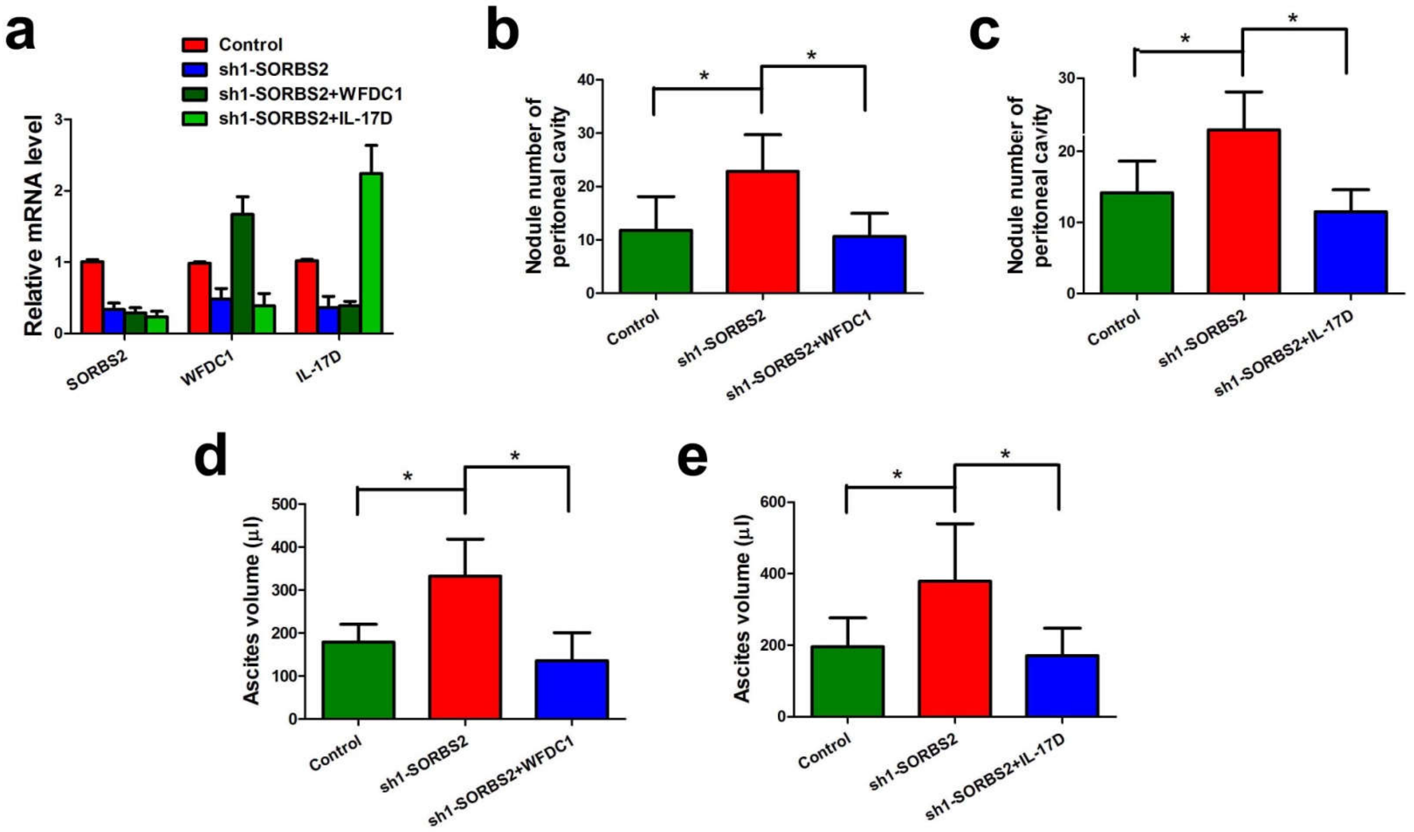

a
Control
sh1-SORBS2
sh1-SORBS2+WFDC1
sh1-SORBS2+IL-17D
Relative mRNA level
SORBS2
WFDC1
IL-17D
b
Nodule number of peritoneal cavity
Control
sh1-SORBS2
sh1-SORBS2+WFDC1
c
Nodule number of peritoneal cavity
Control
sh1-SORBS2
sh1-SORBS2+IL-17D
d
Ascites volume (μl)
Control
sh1-SORBS2
sh1-SORBS2+WFDC1
e
Ascites volume (μl)
Control
sh1-SORBS2
sh1-SORBS2+IL-17D

Supplementary Figure Legends

Supplementary Figure 1. Kaplan Meir analysis of the expression of BTF3, CIRBP and MEX3D and clinical outcome of ovarian cancer in AOCS dataset.

Supplementary Figure 2.

a. SORBS2 expression in normal ovarian tissues, borderline ovarian tumor tissues and ovarian cancer tissues of datasets in Oncomine database. **b.** SORBS2 expression in different clinical stages of ovarian cancer datasets in Oncomine database. **c.** Immunohistochemistry analysis of SORBS2 expression in normal ovarian tissues and ovarian cancer tissues. **d.** Kaplan Meir analysis of SORBS2 expression and clinical outcome of ovarian cancer in West China Ovarian Cancer cohort.

Supplementary Figure 3. SORBS2 suppresses ovarian cancer aggressiveness in clinical samples.

The expression of BTF3, CIRBP, and MEX3D in primary and metastatic ovarian cancer tissues of datasets in Oncomine database.

Supplementary Figure 4. CSIOVDB analysis of SORBS2 expression in ovarian cancer.

a. SORBS2 expression in normal ovary surface epithelium(OSE) and ovarian tumors in CSIOVDB database. **b.** SORBS2 expression in specimens of ovarian cancer with different differentiation grades. **c.** SORBS2 expression in specimens of ovarian cancer with different FIGO stages. **d**. SORBS2 expression in specimens of chemotherapy-sensitive, resistant and refractory ovarian cancer. **e**. Kaplan–Meier analysis of ovarian cancer patients in CSIOVDB database for the correlation between SORBS2 and disease free survival. **f.** Kaplan–Meier analysis of ovarian cancer patients in CSIOVDB database for the correlation between SORBS2 and overall survival. Data are shown as mean ±SEM. *, $P < 0.05$; **, $P < 0.01$; ***, $P < 0.001$.

Supplementary Figure 5. The correlation of SORBS2 expression with disease stage and age in Tothill dataset. a. The expression of SORBS2 expression in stage I-IV ovarian cancer patients. **b.** Correlation data for SORBS2 versus age in Tothill dataset. The statistical significance of correlations was determined using Pearson's

correlation coefficient. Data are shown as mean ±SEM. *, $P < 0.05$; **, $P < 0.01$; ***, $P < 0.001$.

Supplementary Figure 6. The expression of SORBS2 in different subtypes of ovarian cancer. a. Expression of SORBS2 in different molecular subtypes of ovarian cancer cell lines. **b.** Expression of SORBS2 in BRCA1 mutant(MUT) and BRCA1 wild type(WT) ovarian cancer tissues in TCGA dataset. **c.** Expression of SORBS2 in BRCA2 mutant(MUT) and BRCA2 wild type(WT) ovarian cancer tissues in TCGA dataset. **d.** Correlation data for SORBS2 versus CCNE1 expression in TCGA dataset. The statistical significance of correlations was determined using Pearson's correlation coefficient. **e.** Expression of SORBS2 in CCNE1 low and CCNE1 high ovarian cancer tissues in TCGA dataset. Data are shown as mean ±SEM. *, $P < 0.05$; **, $P < 0.01$; ***, $P < 0.001$.

Supplementary Figure 7.

a. mRNA levels of SORBS2 in SORBS2-knock down and control A2780s ovarian cancer cells. **b.** mRNA levels of SORBS2 in SORBS2-knock down and control SKOV-3 ovarian cancer cells. **c**. Protein levels of SORBS2 and Flag in A2780s ovarian cancer cells stably transfected with Flag-SORBS2 plasmid and control. Data are shown as mean ±SEM. *, $P < 0.05$; **, $P < 0.01$; ***, $P < 0.001$.

Supplementary Figure 8. The impact of SORBS2 depletion on cellular proliferation, apoptosis, cellular morphology and cell cycle *in vitro*. a. Immunohistochemistry analysis of Ki-67 in the tumor tissues of A2780s ovarian cancer cells expressing one of the two independent shRNAs targeting SORBS2 or a control shRNA. **b.** Protein level of cleaved caspase 3 in the lysed tumor tissues of A2780s ovarian cancer cells expressing one of the two independent shRNAs targeting SORBS2 or a control shRNA. **c.** The relative percentage of PI/Annexin V positive cells in the lysed tumor tissues of A2780s ovarian cancer cells expressing one of the two independent shRNAs targeting SORBS2 or a control shRNA. **d. The** representative microscopic photographs of SORBS2-knock down and control A2780s ovarian cancer cells. **e.** Cell proliferation of A2780s and SKOV-3 cells expressing shRNAs targeting SORBS2 or a control shRNA was assessed by colony formation assay. **f.** The percentage of different phases of cell cycles in SORBS2-knock down

and control A2780s and SKOV-3 ovarian cancer cells. Data are shown as mean ±SEM. *, $P < 0.05$; **, $P < 0.01$; ***, $P < 0.001$.

Supplementary Figure 9.

a. Transcripts bound by SORBS2 identified by RIP sequencing. IgG was used as negative control. **b**. Heatmap analysis showing RIP sequencing data for SORBS2 and IgG control at a 20-kb region centered over TSS. **c.** SORBS2 RIP-seq read density mapped onto the 3′ UTRs of *WFDC1* and *IL-17D*.

Supplementary Figure 10.

a. qRT-PCR of candidate SORBS2 target transcripts in A2780s shSORBS2 and shCTRL cells at steady-state. HPRT1 was used as an endogenous control. **b.** qRT-PCR of candidate SORBS2 target transcripts in SKOV-3 shSORBS2 and shCTRL cells at steady-state. HPRT1 was used as an endogenous control. **c.** qRT-PCR for increasingly distal regions of the *WFDC1* transcript 3'UTRs relative to the level of the CDS of each of the genes in A2780s shCTRL and shSORBS2 cells. **d.** qRT-PCR for increasingly distal regions of the *IL-17D* transcript 3'UTRs relative to the level of the CDS of each of the genes in A2780s shCTRL and shSORBS2 cells. **e**. Western blots showing levels of WFDC1 and IL-17D proteins in A2780s shCTRL and shSORBS2 whole cell lysate. Biological triplicates are shown. β-actin was used as a loading control. **f.** Relative concentration of WFDC1 and IL-17D in the conditioned media of A2780s shCTRL and shSORBS2 cells. Data are shown as mean ±SEM. *, $P < 0.05$; **, $P < 0.01$; ***, $P < 0.001$.

Supplementary Figure 11. The impact of enhanced SORBS2 expression in the transcript stability and 3' UTR lengths of *WFDC1* and *IL-17D*. a. qRT-PCR of relative WFDC1 mRNA level in SORBS2-overexpressing and control A2780s cells at the times indicated after treatment of cells with DRB. **b.** qRT-PCR of relative IL-17D mRNA level in SORBS2-overexpressing and control A2780s cells at the times indicated after treatment of cells with DRB. **c.** qRT-PCR for increasingly distal regions of the *WFDC1* transcript 3'UTRs relative to the level of the CDS of each of the genes in SORBS2-overexpressing and control A2780s cells. **d.** qRT-PCR for increasingly distal regions of the *IL-17D* transcript 3'UTRs relative to the level of the CDS of each of the genes in SORBS2-overexpressing and control A2780s cells. Data

are shown as mean ±SEM. *, $P < 0.05$; **, $P < 0.01$; ***, $P < 0.001$.

Supplementary Figure 12.

a. Immunohistochemistry analysis of WFDC1 and IL-17D expression in SORBS2-low and SORBS2-high primary tissues of ovarian cancer. **b.** The relative concentration of WFDC1 and IL-17D in the ascites of ovarian cancer patients in low and high SORBS2 tissues. Data are shown as mean ±SEM. *, $P < 0.05$; **, $P < 0.01$; ***, $P < 0.001$.

Supplementary Figure 13.

a. The relative mRNA levels of WFDC1 in A2780s cells stably expressing shCTRL+vector, sh1-SORBS2+vector, sh1-SORBS2+WFDC1, sh1-SORBS2+IL-17D, and sh1-SORBS2+WFDC1/IL-17D. **b.** The relative mRNA levels of IL-17D in A2780s cells stably expressing shCTRL+vector, sh1-SORBS2+vector, sh1-SORBS2+WFDC1, sh1-SORBS2+IL-17D, and sh1-SORBS2+WFDC1/IL-17D. **c.** Box plot of number of metastatic nodules of tumors in the abdominal cavities of mice inoculated with A2780s cells stably expressing shCTRL+vector, sh1-SORBS2+vector, sh1-SORBS2+WFDC1, sh1-SORBS2+IL-17D, and sh1-SORBS2+WFDC1/IL-17D. **d.** Box plot of number of metastatic nodules of tumors in the abdominal cavities of mice inoculated with A2780s cells stably expressing shCTRL+vector, sh1-SORBS2+vector, sh1-SORBS2+WFDC1, sh1-SORBS2+IL-17D, and sh1-SORBS2+WFDC1/IL-17D. Data are shown as mean ±SEM. *, $P < 0.05$; **, $P < 0.01$; ***, $P < 0.001$.

Supplementary Figure 14.

a. The transfection efficacy was evaluated by measuring the mRNA levels of each gene in each group. **b.** The number of metastatic nodules of C57BL/6 mice model intrabursally inoculated with control ID-8 cells, SORBS2-knockdown ID-8 cells and WFDC1 overexpressing SORBS2-knockdown ID-8 cells at sacrifice. n=6 in each group. **c.** The number of metastatic nodules of C57BL/6 mice model intrabursally inoculated with control ID-8 cells, SORBS2-knockdown ID-8 cells and IL-17D overexpressing SORBS2-knockdown ID-8 cells at sacrifice. n=6 in each group. **d.** The ascites volume of C57BL/6 mice model intrabursally inoculated with control ID-8 cells, SORBS2-knockdown ID-8 cells and WFDC1 overexpressing

SORBS2-knockdown ID-8 cells at sacrifice. n=6 in each group. **e.** The ascites volume of C57BL/6 mice model intrabursally inoculated with control ID-8 cells, SORBS2-knockdown ID-8 cells and IL-17D overexpressing SORBS2-knockdown ID-8 cells at sacrifice. n=6 in each group. Data are shown as mean ±SEM. *, $P < 0.05$; **, $P < 0.01$; ***, $P < 0.001$.
